# An unprecedented palladium-arsenic catalytic cycle for nitriles hydration

**DOI:** 10.3389/fchem.2023.1253008

**Published:** 2023-08-07

**Authors:** Damiano Cirri, Tiziano Marzo, Alessandro Pratesi

**Affiliations:** ^1^ Department of Chemistry and Industrial Chemistry, University of Pisa, Pisa, Italy; ^2^ Department of Pharmacy, University of Pisa, Pisa, Italy

**Keywords:** nitrile hydration, palladium catalysis, arsenic catalysis, heterobimetallic catalysis, amide synthesis

## Abstract

An unprecedented palladium/arsenic-based catalytic cycle for the hydration of nitriles to the corresponding amides is here described. It occurs in exceptionally mild conditions such as neutral pH and moderate temperature (60°C). The versatility of this new catalytic cycle was tested on various nitriles from aliphatic to aromatic. Also, the effect of ring substitution with electron withdrawing and electron donating groups was investigated in the cases of aromatic nitriles, as well as the effect of potentially interferent functional groups such as hydroxy group or pyridinic nitrogen. Furthermore, a pilot study on the potential suitability of this approach for its scale-up is presented, revealing that the catalytic cycle could be potentially and quickly scaled up.

## 1 Introduction

The amide function undoubtedly represents one of the most important groups in several fields of chemistry. The peptide bond is indeed a type of amide chemical bond allowing to exert the key functions of proteins and in turn the chemistry of life (Goswami and Van Lanen, 2015; Mahesh et al., 2018). Pairwise, amides are “building bricks” for several technological and organic, industrial, pharmaceutical, and material chemistry applications. Amide groups are commonly found in high-value industrial chemicals, e.g., polymers, plasticizers, solvents (Le Berre et al., 2014), and lubricants (Khalkar et al., 2013). For this reason, the search for improved processes for amides production is of great interest to the chemical industry (Messa et al., 2018). Several synthetic routes are exploitable in the case of laboratory-scale; however, most of them imply drastic experimental conditions that may not be tolerated by other functional groups present on the molecule itself (Montalbetti and Falque, 2005). Moreover, these methods typically suffer from a low atom economy and are associated with the generation of large quantities of waste products, making them scarcely sustainable (Ojeda and Yilmaz, 2013; Hahladakis et al., 2018). In large-scale industrial production, the nitrile hydration process is surely a forced choice due to the more favorable atom economy. Unfortunately, also this latter approach has some relevant drawbacks since the hydration reaction is usually catalyzed by strongly acidic or basic conditions that may result in a lack of fine reaction control and of the stability of the amides themselves. Moreover, owing to the harsh reaction conditions, the accurate control of the hydration process is frequently unsatisfactory. Indeed, on the one hand, in the case of basic catalysis the desired amide is often hydrolyzed to the corresponding carboxylate with stoichiometric consumption of the catalyst. On the other hand, in the case of an acid-mediated process, careful control of the temperature and of the reagents’ ratio is required to avoid the occurrence of a polymerization process triggered by the exothermic character of the hydrolysis reaction (Kukushkin and Pombeiro, 2005). The formation of side products, such as neutralization salts, is an additional drawback that imposes burdensome problems in the disposal of chemical waste. Accordingly, there is a strong interest in discovering new and more efficient amide production strategies capable to overcome the limitations of the currently available procedures, especially at the industrial scale. In this frame, some interesting results have been obtained by Parkins and coworkers using platinum phosphinito catalysts, but the described processes required reaction temperatures ≥90°C (Ghaffar and Parkins, 1995; 2000; Parkins, 1996). Moreover, some other methods have been described using metallic or enzymatic catalysts (García-Álvarez et al., 2014; Tomás-Mendivil et al., 2014; Bryan et al., 2018; Messa et al., 2018; Cheng et al., 2020; Alcántara et al., 2022). Anyway, to the best of our knowledge, these latter alternative approaches still suffer from relevant complications, such as the employment of carbon monoxide (Messa et al., 2018), the need for precious metals-containing coordination complexes as catalysts, or enzymes with low thermal stability and narrow substrate tolerability (García-Álvarez et al., 2014; Tomás-Mendivil et al., 2014; Bryan et al., 2018; Cheng et al., 2020; Alcántara et al., 2022). As a contribution in this direction, we describe here a novel nitriles hydration process for the convenient conversion into the respective amides, based on a palladium/arsenic-mediated catalytic cycle. This catalytic hydration process uses commercially available catalysts, such as palladium chloride and arsenic trioxide. Moreover, the catalytic cycle is effective at neutral pH and moderate temperature (60°C). Due to these mild conditions, no by-products are generated, and the unreacted nitrile can be fully recovered through classical procedures such as distillation or flash column chromatography. This latter aspect is highly desirable in laboratory-scale fine chemistry research, where the substrate might be a expensive custom-synthesized molecule. The experimental conditions have been derived after several attempts. More precisely, in the case of water-immiscible nitriles, DMF turned out to be the only effective cosolvent (others, such as THF, acetone and ethanol, did not allow the reaction to work at all). Based on these data we pointed out that, overall, the ideal cosolvent must ensure the complete solubilization of the substrate and at the same time must be non-coordinating towards the Pd center because coordination hampers the catalytic cycle. Additionally, DMF is non-protic and has the required polarity (compared with other tested non-protic solvents such as acetone). Also, a wide range of temperatures has been explored: as a rule of thumb, higher temperatures speed up the hydration process together with catalyst deactivation. The temperature of 60°C turned out to be a good compromise among these two contrasting effects, moreover, in the case of hydrophobic nitriles, prevents substrate precipitation.

## 2 Materials and methods

Four different experimental setups were explored, namely, A, B, C and D. These four procedures were used for liquid and water-miscible nitriles (A), liquid hydrophobic nitriles (B) and solid nitriles (C and D), see Table 1. All setups have been optimized for keeping substrates in solution with as little amount of DMF as possible. For comparison purposes between the different setups, it should be taken into account that, in order to avoid experimental bias (due to the extremely small amount of catalysts it would have been necessary to weigh), a larger amount of catalysts has been used in for setup A-D (0.050 mmol and 0.075 mmol for PdCl_2_ and As_2_O_3_, roughly 1:380 with respect to substrates). At variance, the amount of PdCl_2_ and As_2_O_3_ has been decreased (ratio 1:6,500, see below) in the large-scale setup allowing the calculation of the TON. For the characterization of the obtained products, the NMR spectra were acquired on a JEOL 400 YH spectrometer (resonating frequencies: 400 and 100 MHz for ^1^H and ^13^C, respectively). Spectra were recorded at room temperature (25°C ± 2°C) using solvents with a deuteration degree ≥99.8% and calibrated on solvent residual signals. Deuterated solvents were purchased from Deutero GmbH.

**TABLE 1:** Experimental hydration yields for the tested substrates.

Substrate	Experimental setup^[^ ^a^ ^]^	% amide yield
Acetonitrile	A	48
Propionitrile	B	61
Methacrylonitrile	B	10
Phenylacetonitrile	B	56
Benzonitrile	B	54
2-Hydroxybenzonitrile	C	43
4-(Dimethylamino)benzonitrile	C	34
4-Bromobenzonitrile	C	70
4-Nitrobenzonitrile	C	94
Benzene-1,2-dicarbonitrile^[^ ^b^ ^]^	C	14
Benzene-1,4-dicarbonitrile^[^ ^b^ ^]^	D	31
3-Pyridinecarbonitrile	C	0
4-Pyridinecarbonitrile	C	0
2-Pyrimidinecarbonitrile	C	0
4-Cyano-1-methylpyridin-1-ium nitrate	C	17^[^ ^c^ ^]^

^a^ Experimental details are provided in the Material and methods section.

^b^ Only monohydrated products are present.

^c^ ^1^H NMR, analysis of the crude product shows quantitative hydration of starting nitrile. The low final yield is due to product loss during the filtration step (over celite), owing to the product’s ionic form.

### 2.1 Setup A (liquid and water-miscible substrates)

1 mL of acetonitrile and 10 mg of palladium chloride were added into a 50 mL flask. The suspension was stirred and heated to 60°C with an oil bath. After 30 min 1 mL of demineralized water and 15 mg of arsenic trioxide were added. The suspension was stirred at 60°C overnight, then the liquid phase in the flask was completely evaporated, and the crude product was purified through classical flash column chromatography eluting with 100% EtOAc.

### 2.2 Setup B (liquid and water-insoluble substrates)

1 mL of nitrile and 10 mg of palladium chloride were added into a 50 mL flask. The suspension was stirred and heated to 60°C with an oil bath. After 30 min 1 mL of demineralized water, 0.5 mL of DMF and 15 mg of arsenic trioxide were added. The suspension was stirred at 60°C overnight, then the liquid phase in the flask was completely evaporated, and the crude products were purified through classical flash column chromatography (100% EtOAc for propionamide and methacrylamide; gradient between CHCl_3_ and EtOAc for phenylacetamide and benzamide).

### 2.3 Setup C (solid and water-insoluble substrates)

600 mg of nitrile, 2 mL of DMF and 10 mg of palladium chloride were added into a 50 mL flask. The suspension was stirred and heated to 60°C with an oil bath. After 30 min 0.5 mL of demineralized water and 15 mg of arsenic trioxide were added. The suspension was stirred at 60°C overnight, then the liquid phase in the flask was completely evaporated, and the crude products were purified through classical flash column chromatography (gradient between CHCl_3_ and EtOAc for 2-cyanobenzamide, 4-dimethylaminobenzamide, 4-bromobenzamide; gradient between CHCl_3_ and acetone for 4-nitrobenzamide; CH_2_Cl_2_/EtOAc 9:1 for 2-hydroxybenzamide) or simple filtration on celite septum after dissolution in methanol (4-cyano-1-methylpyridin-1-ium nitrate).

### 2.4 Setup D (solid and extremely water-insoluble substrates)

This experimental procedure can be seen as a modification of setup C and it was explored due to the low solubility of benzene-1,4-dicarbonitrile, which did not dissolve in the condition described in setup C. 640 mg of nitrile, 4 mL of DMF and 10 mg of palladium chloride were added into a 50 mL flask. The suspension was stirred and heated to 60°C with an oil bath. After 30 min 1 mL of demineralized water and 15 mg of arsenic trioxide were added. The suspension was stirred at 60°C overnight, then the liquid phase in the flask was completely evaporated, and the crude product was purified through classical flash column chromatography with a gradient between CHCl_3_ and EtOAc.

### 2.5 Large scale setup 1

170 mL of acetonitrile (132.6 g; 3.210 mol), 178 mg of palladium chloride (1.00 mmol), 198 mg of arsenic trioxide (1.00 mmol) and 60 mL of demineralized water were added into a 1 L flask. The suspension was refluxed overnight at 110°C in an oil bath. After that, the suspension was left to cool and subsequently filtered for removing the formed metal particles. The resulting solution was reduced in volume through a rotary evaporator and then dried under vacuum until the formed acetamide turned out to be completely dried. With this setup were recovered 76 g of acetamide; 41% yield (in good agreement with the calculated one in setup A).

### 2.6 Large scale setup 2

This experiment was performed with the same modalities as above, but with a reduction of the catalytic materials to 90 mg of palladium chloride (0.50 mmol) and 95 mg of arsenic trioxide (0.50 mmol). With this setup, we experienced a yield reduction to 30%, which allowed us to calculate the catalyst turnover number. TON: 1864.

Acetamide obtained in both large-scale setups was purified only through filtration since these experiments served only for method robustness confirmation and TON calculation.

## 3 Results and discussion

The likely catalytic cycle is described and discussed together with a preliminary scale-up attempt focused on a simple and industrially relevant substrate such as acetonitrile. A relationship in terms of substrate structure and reaction yields is also provided. The hydration process was conducted on sixteen different substrates, whose results are summarized in Table 1. The selection of substrates covered a wide range of cases, spreading from aliphatic to aromatic nitriles. In addition, the effects of ring substitution with EWG and EDG groups have been investigated in the cases of aromatic nitriles, as well as the effect of potentially interferent functional groups such as hydroxy group or pyridinic nitrogen.

**FIGURE 1:**
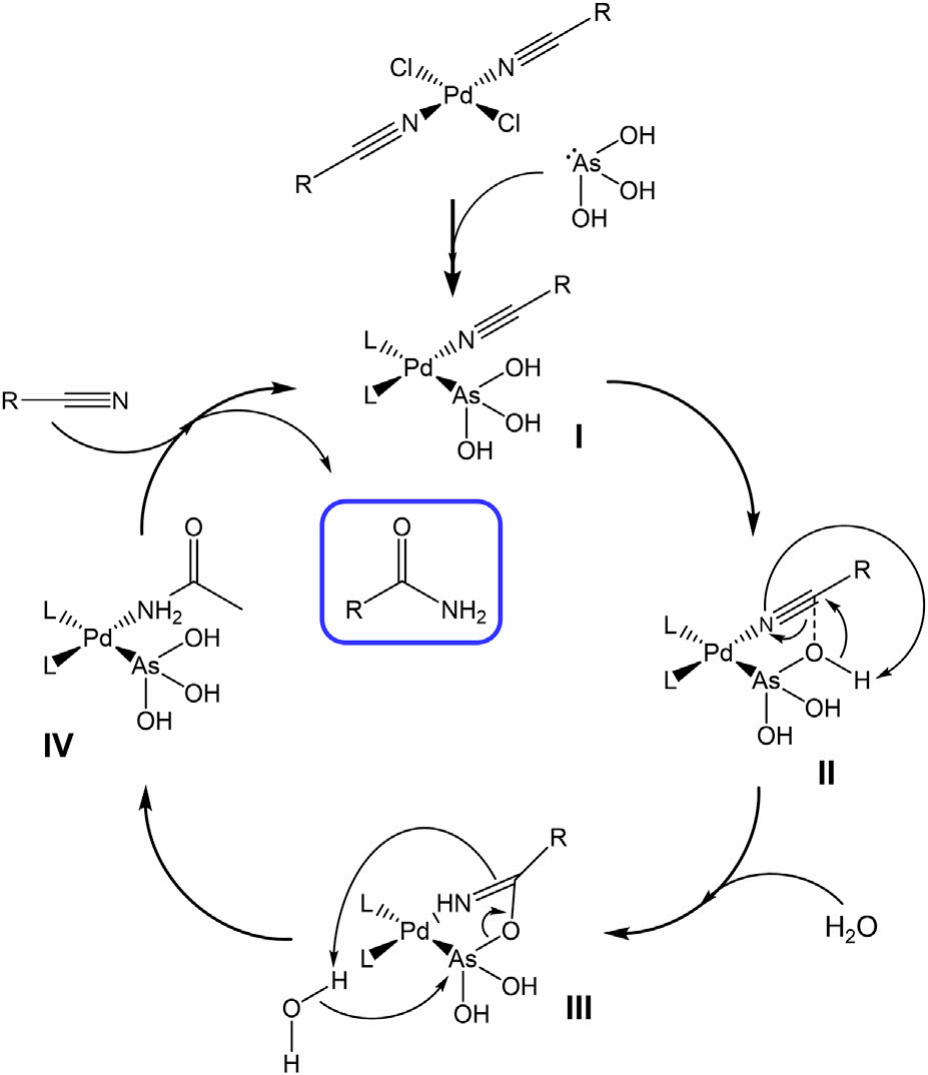
Proposed mechanism for the catalytic cycle.

The reaction takes place as follows: a catalytic amount of PdCl_2_ is reacted with the selected nitrile to afford the square planar complex bis(nitrile)dichloropalladium(II). Subsequently, an excess of water is added together with As_2_O_3_. In these conditions, As_2_O_3_ reacts with water to form the unstable arsenous acid. This latter, owing to the availability of the arsenic electron lone pair, acts as a ligand towards the palladium center. Therefore, we presume the formation of a catalytic species in which the palladium atom holds a nitrile molecule in the spatial proximity of an arsorous acid moiety (Figure 1, step I). As depicted in Figure 1, we assume that the arsenic atom bound to hydroxyl groups acts as a nucleophile toward the palladium-coordinated nitrile carbon atom (step II). Then, the reaction proceeds with the intervention of a water molecule (as depicted in step III) causing the opening of the five-membered ring and the regeneration of the arsorous acid moiety that still remains coordinated to the Pd(II) center (as depicted in Figure 1, step IV). The release of the formed amide and the coordination of a new nitrile molecule to the Pd(II) atom complete the catalytic cycle.

Our mechanistic hypothesis is supported by the following evidence.

- The reaction is not effective in the presence of palladium chloride or arsenic trioxide alone. The hydration takes place only if both catalysts are present in the reaction environment.
- Examples exist concerning the Pd-As(OH)_3_ direct bond, as well as the occurrence of Pt-As motifs. More precisely, coordination bonds have been already reported in the literature and described through robust experimental approaches including X-Ray crystallography (Sokolov et al., 2001; Miodragović et al., 2013).
- The reaction is not effective toward substrates endowed with stronger coordinating groups than nitrile moiety. In those cases, there is a competition for the coordination to the palladium center and this behavior has been verified with pyridines, pyrimidines, or cationic substrates with a coordinating iodide as the counterion. This aspect suggests that coordination of the nitrile group on the palladium atom is a mandatory step for the initiation of the catalytic cycle (Rajput et al., 2004).
- The reaction is highly promoted by the presence of electron-withdrawing groups (EWG) conjugated to the nitrile moiety such as the nitro or N-Methylpiridinium groups (see note [c] of Table. 1). This latter aspect can be easily rationalized in step II (Figure 1) of the proposed reaction mechanism, in which the nucleophilic attack of the oxygen on the nitrile carbon atom is favored by a higher positive density charge induced from EWG substituents.
- The intermediate obtained in step III (Figure 1) can be isolated upon substitution of palladium with a less reactive metal centre (Miodragović et al., 2013).

**FIGURE 2:**
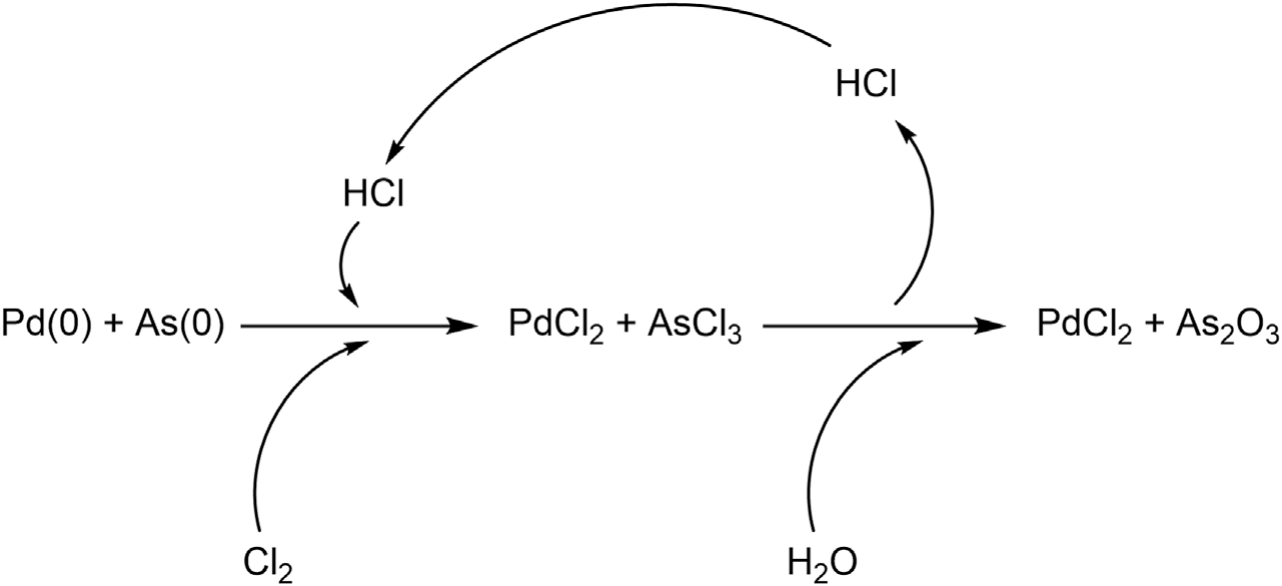
Reactions for the regeneration of the catalytic system.

To note, the catalytic system, which during the reaction may be subject to deactivation due to the reduction, can be regenerated by dissolving the exhausted catalytic mixture with HCl in the presence of Cl_2_ as depicted in Figure 2 (Grund et al., 2008). Under these conditions, Pd(0) can be regenerated to PdCl_2_; while As(0) is converted to AsCl_3_ which is a volatile liquid compound. Accordingly, the separation of the AsCl_3_ from PdCl_2_ can be afforded through a simple distillation process (Nikolashin et al., 2007). A final hydrolysis step of AsCl_3_ will then afford arsenic trioxide. This latter aspect is relevant because significantly enhances the sustainability of the approach, even when scaled up.

Spurred by these interesting results, we have tried to scale up the acetonitrile hydration reaction to assess its suitability for a larger amount of substrate and to estimate the turnover number (TON) of the catalytic arseno-palladium complex. We have therefore performed two large-scale experiments (details are reported in supporting material). In the first large-scale reaction, we confirmed a moderate yield (41% similarly to the 48% of the smaller setup), while in the second experiment we determined the turnover number of the catalytic system, which was calculated to be 1.86×10^3^. This TON is superior to that already reported for catalytic complexes operating in similar mild conditions (Guo et al., 2019).

## 4 Conclusion

In conclusion, we have here reported an unprecedented hydration procedure that might be very useful for the obtainment of aliphatic and aromatic amides from the parent nitriles. Moreover, this procedure has a sustainable profile because it operates with commercially available catalysts in mild conditions, and in the presence of atmospheric oxygen.

## Data Availability

The original contributions presented in the study are included in the article/Supplementary Material, further inquiries can be directed to the corresponding authors.
