# Supplementary material for: An unprecedented palladium-arsenic catalytic cycle for nitriles hydration
Source: Front Chem. 2023 Aug 7;11:1253008. doi: 10.3389/fchem.2023.1253008 (PMC10440694; doi:10.3389/fchem.2023.1253008)
Supplement: Supplementary file 1 [file DataSheet1.docx]

**An unprecedented palladium-arsenic catalytic cycle for nitriles hydration**

Damiano Cirri,^a^ Tiziano Marzo* ^b^ and Alessandro Pratesi* ^a^

Contents

[_Toc100772276](#_Toc100772276)

[Acetamide 2](#_Toc100772277)

[Propanamide 3](#_Toc100772278)

[Methacrylamide 4](#_Toc100772279)

[Phenylacetamide 5](#_Toc100772280)

[Benzamide 6](#_Toc100772281)

[2-Hydroxybenzamide 7](#_Toc100772282)

[4-Dimethylamidobenzamide 8](#_Toc100772283)

[4-Bromobenzamide 9](#_Toc100772284)

[4-Nitrobenzamide 10](#_Toc100772285)

[2-Cyanobenzamide 11](#_Toc100772286)

[4-Cyanobenzamide 12](#_Toc100772287)

[4-Carbamoyl-1-methylpyridin-1-ium nitrate 13](#_Toc100772288)

# NMR spectra of the obtained products

## Acetamide


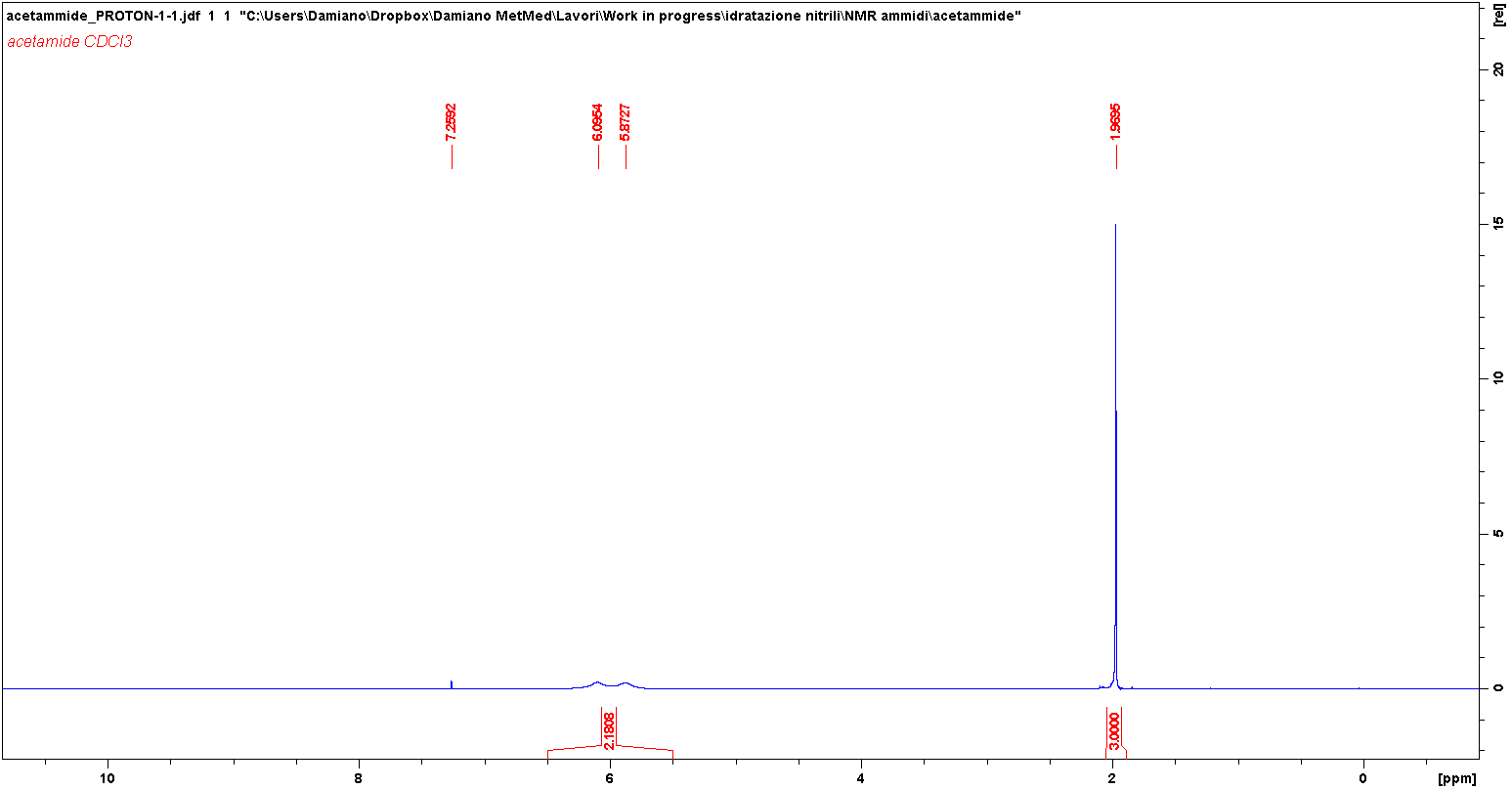


**Fig. S1.** Acetamide ^1^H NMR spectrum (500 MHz; CDCl_3_) δ: 6.09 (1H, b); 5.87 (1H, b); 1.97 (3H, s).


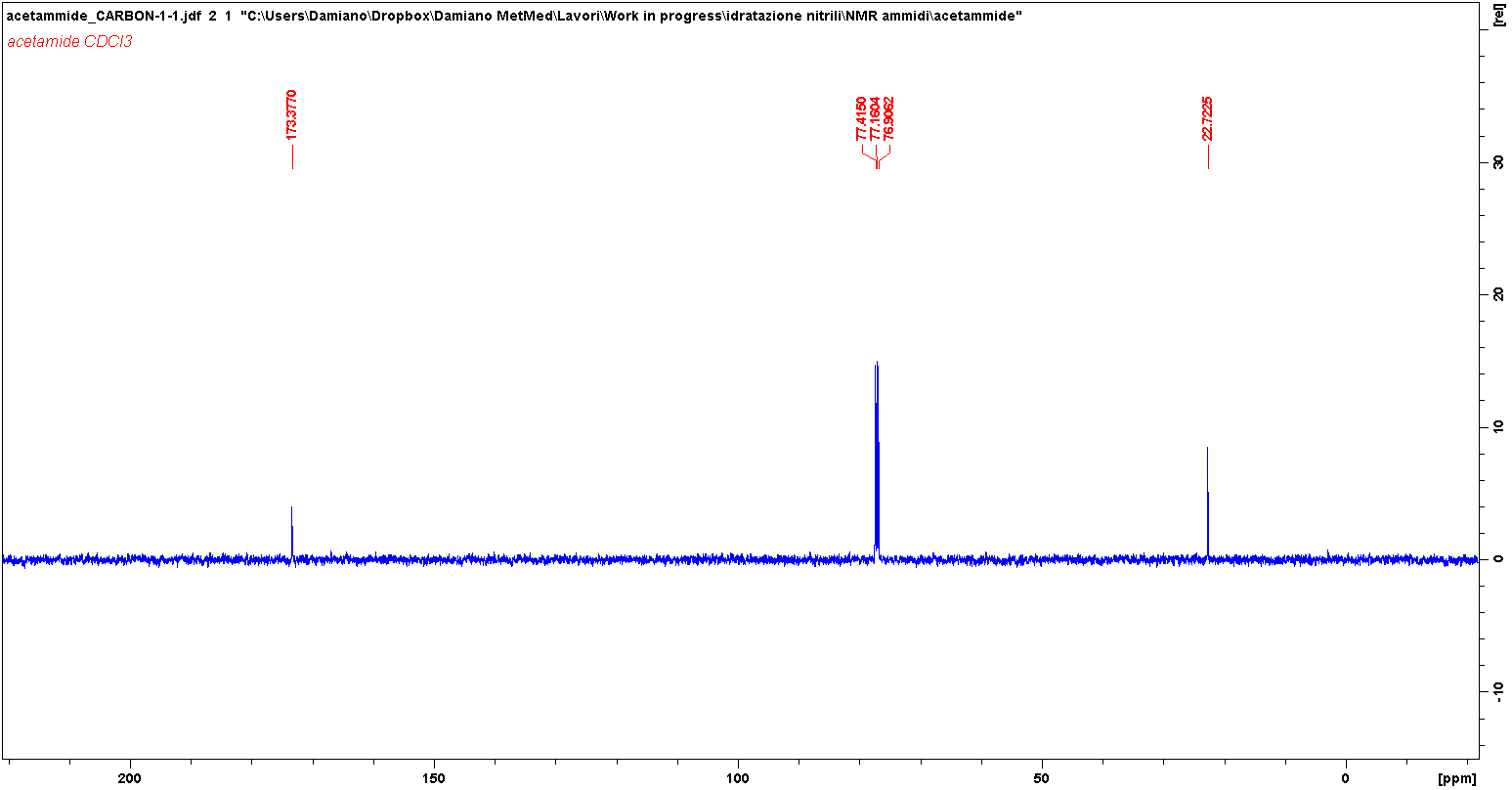


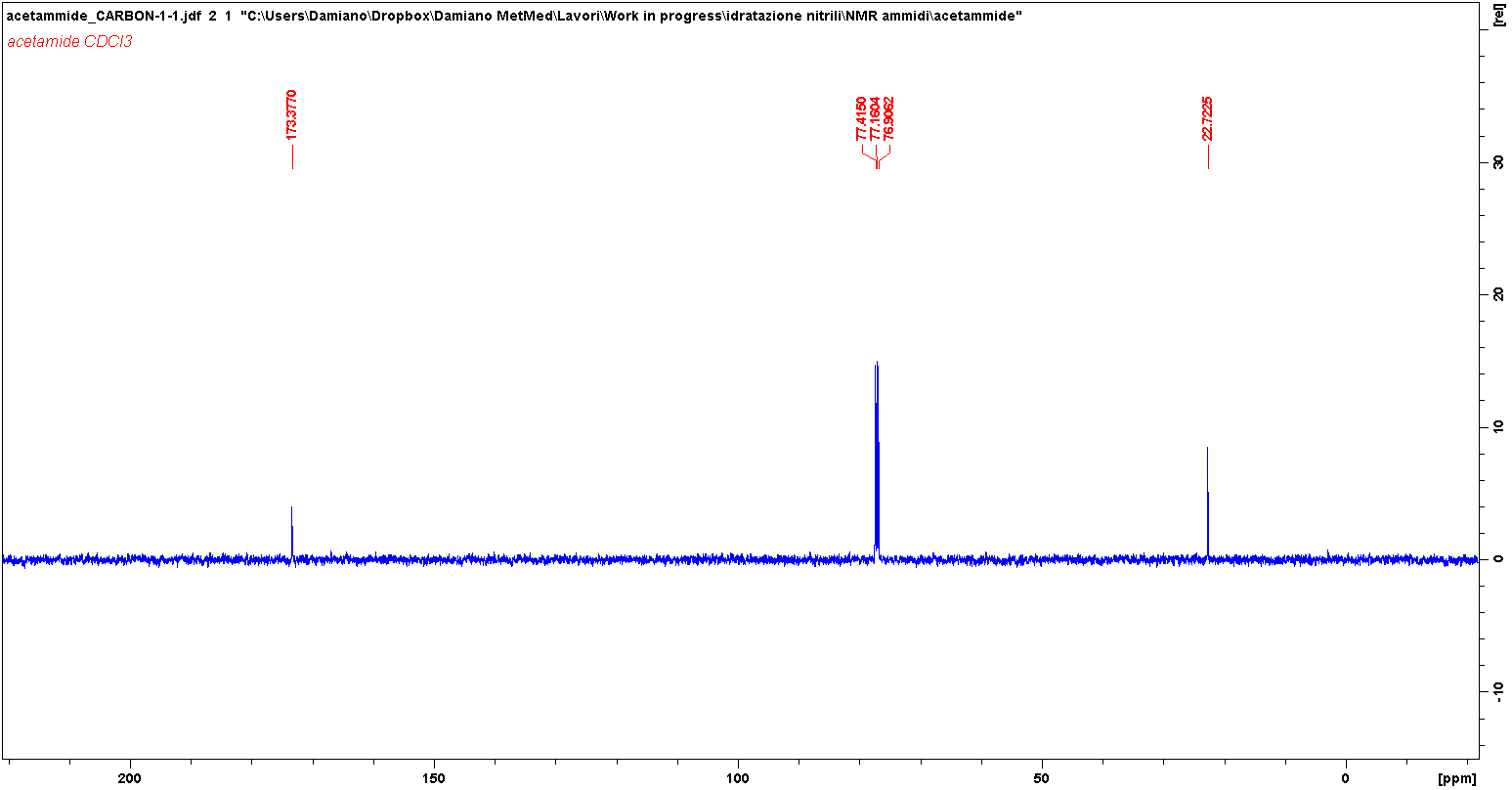
**Fig. S2.** Acetamide ^13^C NMR spectrum (125 MHz; CDCl_3_) δ: 173.4; 22.7.

## Propanamide


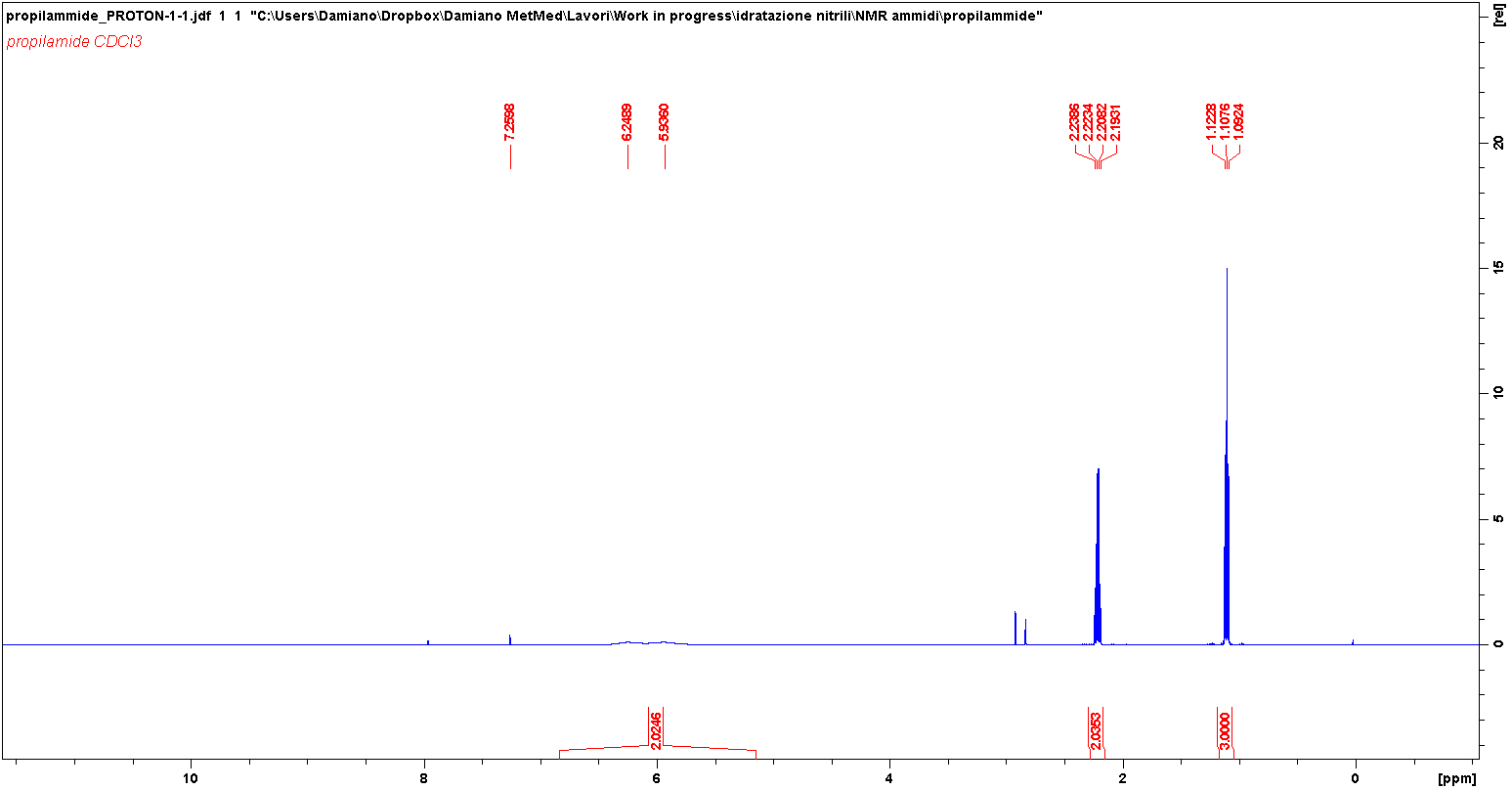


**Fig. S3.** Propanamide ^1^H NMR spectrum (500 MHz; CDCl_3_) δ: 6.25 (1H, b); 5.94 (1H, b); 2.21 (2H, q, *J*= 7.59 Hz); 1.11 (3H, t, *J*= 7.59 Hz).


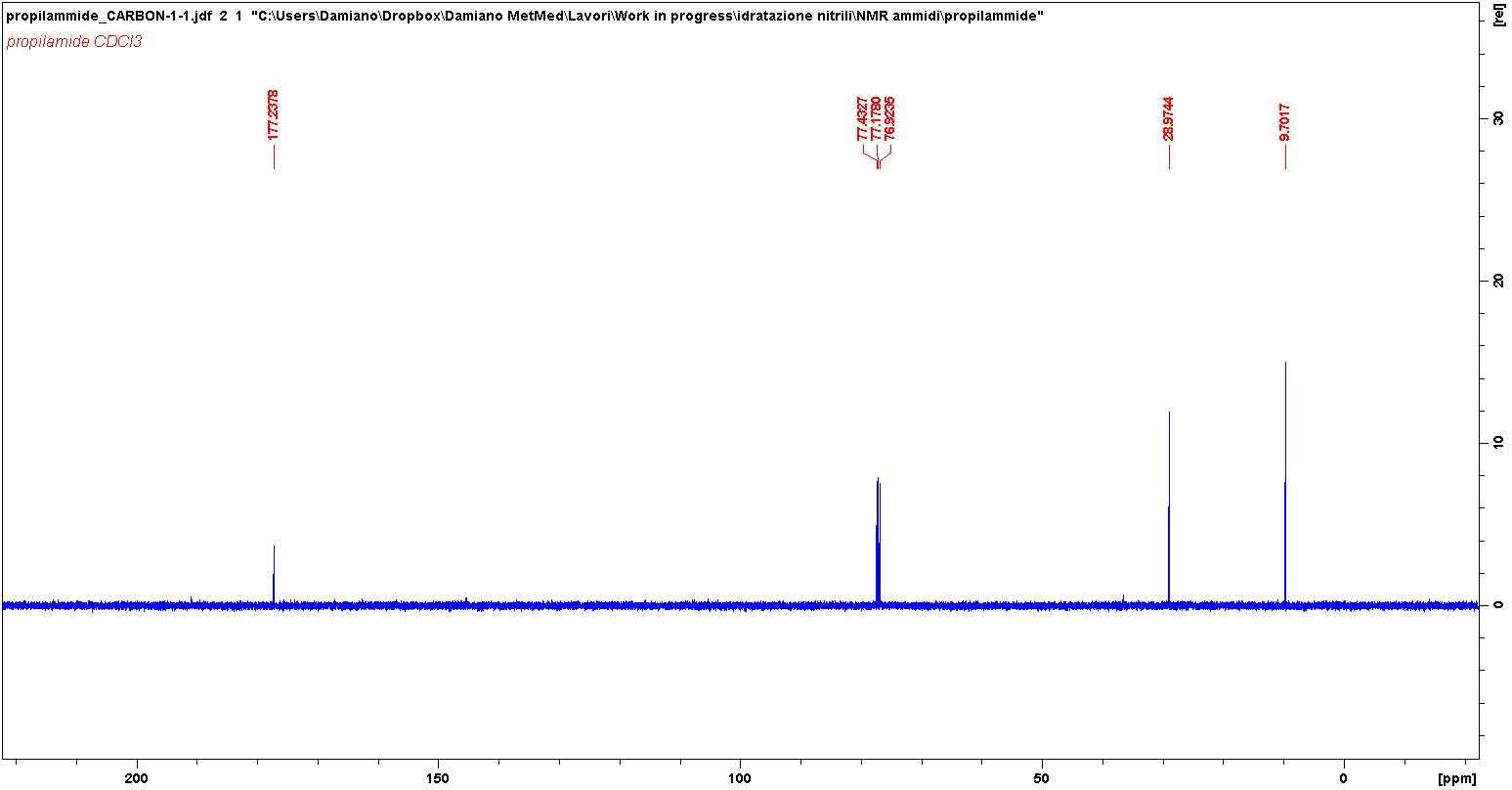


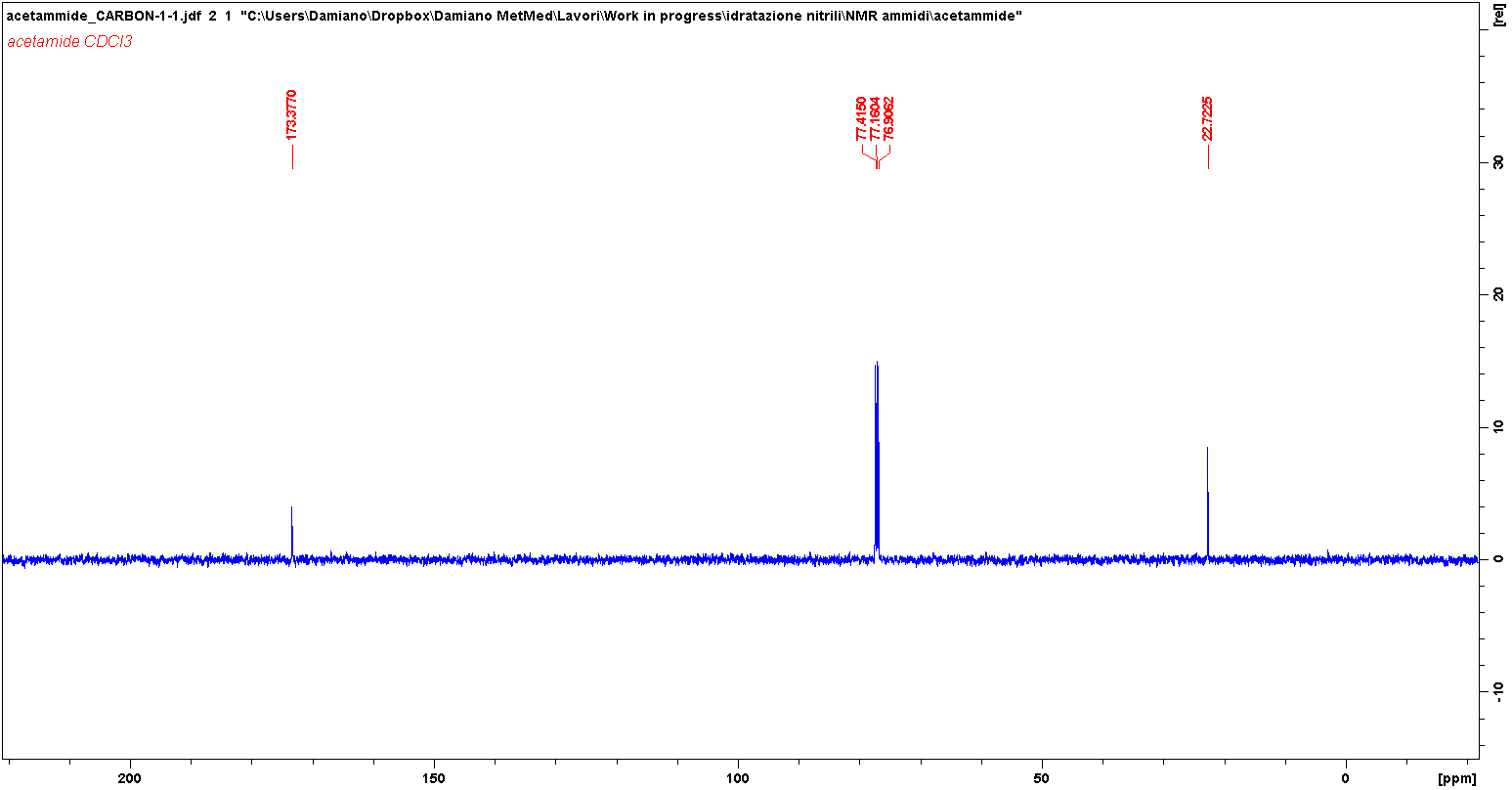
**Fig. S4.** Propanamide ^13^C NMR spectrum (125 MHz; CDCl_3_) δ: 177.2; 28.9; 9.7.

## Methacrylamide


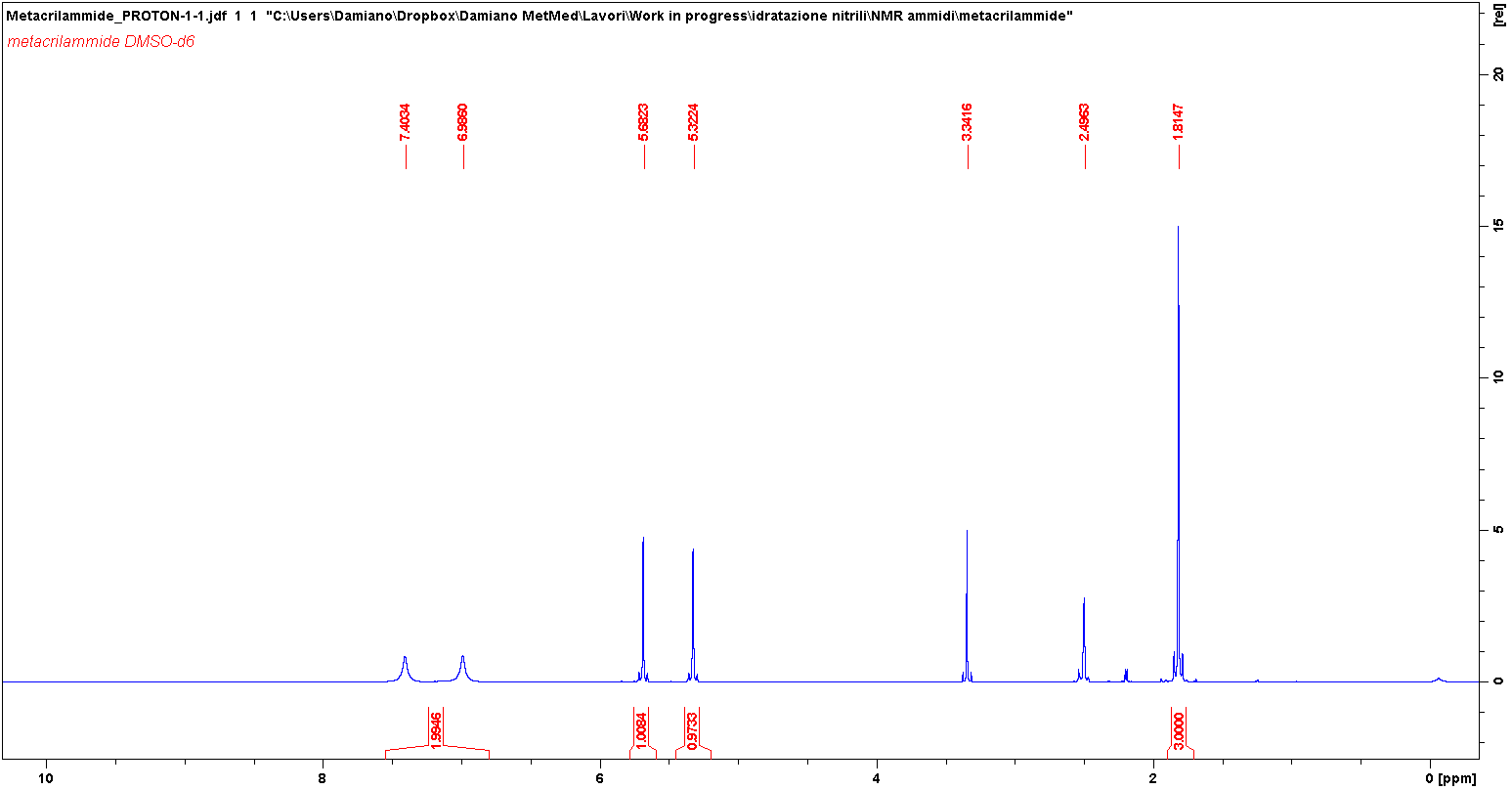


**Fig. S5.** Methacrylamide ^1^H NMR spectrum (500 MHz; DMSO-d_6_) δ: 7.40 (1H, b); 6.99 (1H, b); 5.68 (1H, s); 5.32 (1H, s); 1.81 (3H, s).


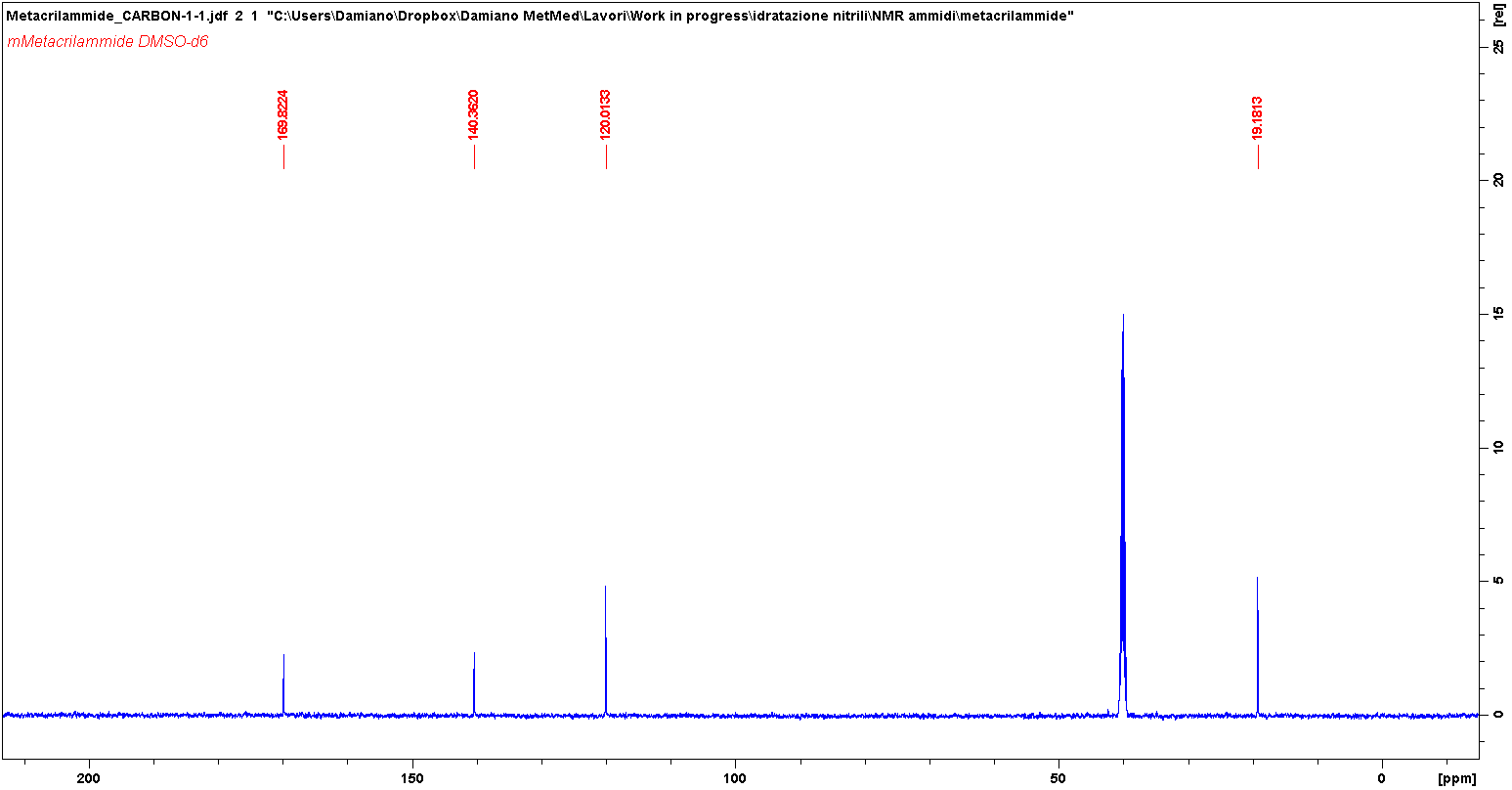


**Fig. S6.** Methacrylamide ^13^C NMR spectrum (125 MHz; DMSO-d_6_) δ: 169.8; 140.4; 120.0; 19.2.

## Phenylacetamide


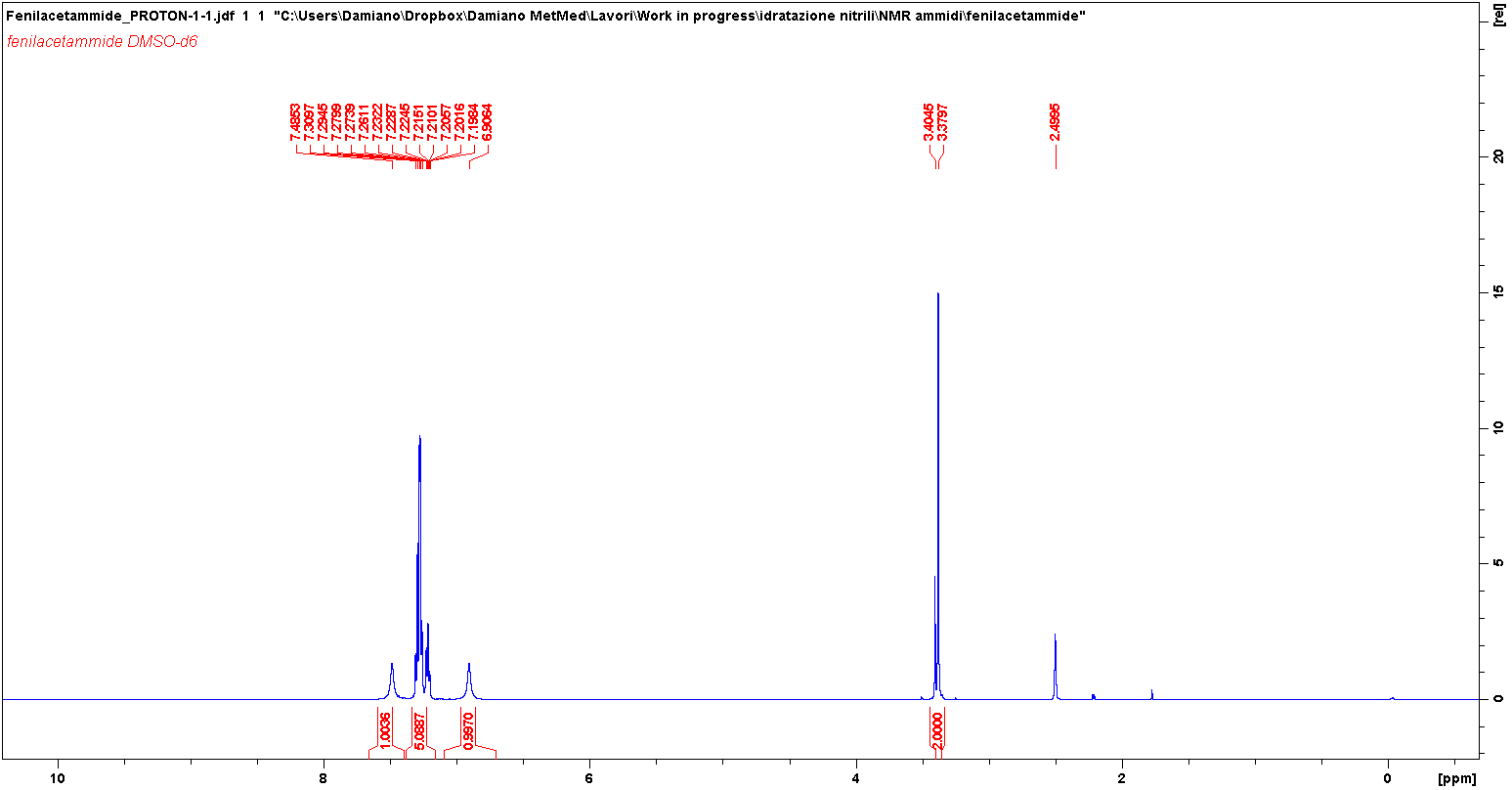


**Fig. S7.** Phenylacetamide ^1^H NMR spectrum (500 MHz; DMSO-d_6_) δ: 7.48 (1H, b); 7.27 (4H, m); 7.21 (1H, m); 6.91 (1H, b); 3.40 (2H, s).


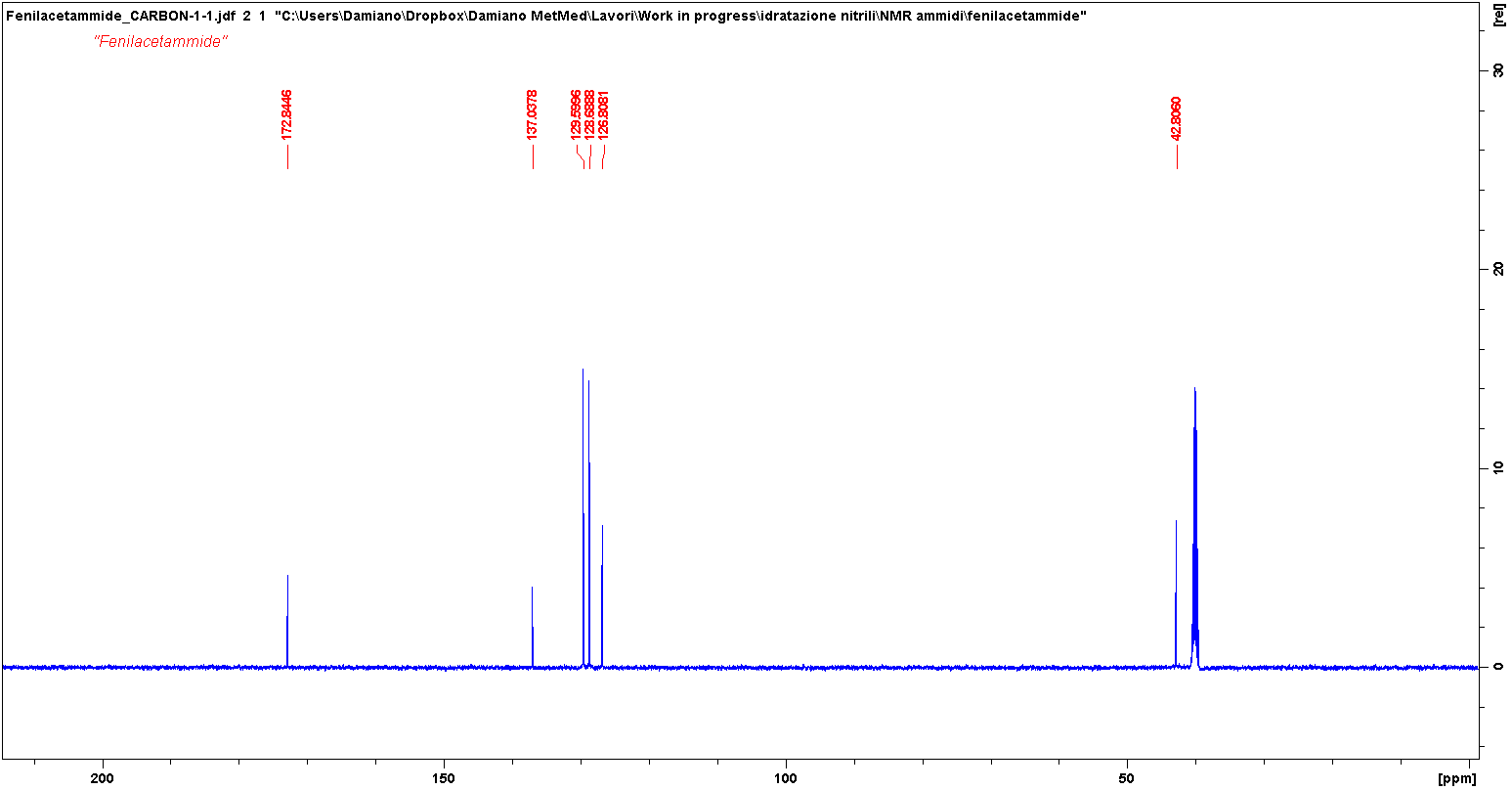


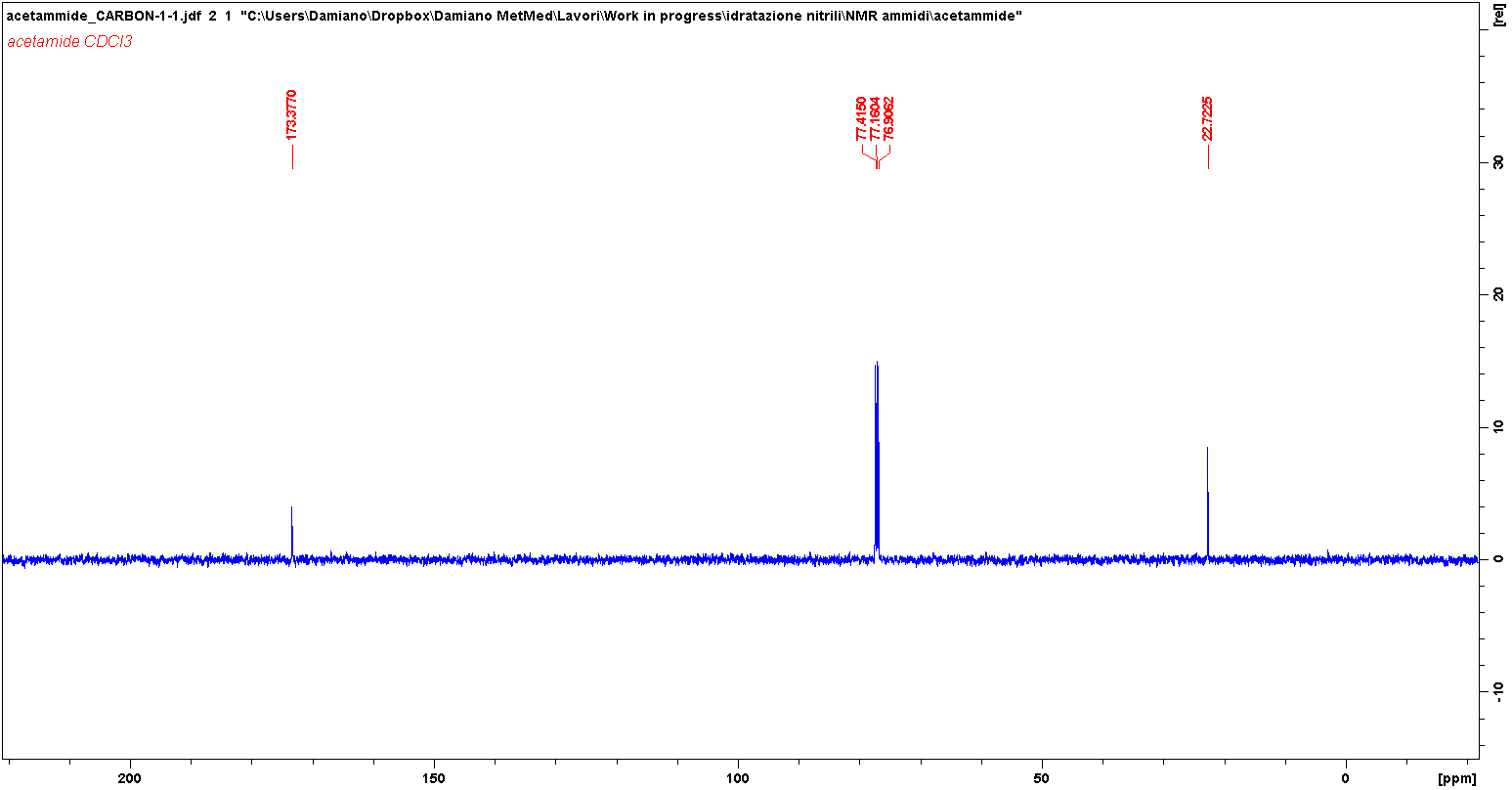
**Fig. S8.** Phenylacetamide ^13^C NMR spectrum (125 MHz; DMSO-d_6_) δ: 172.8; 137.0; 129.6; 128.7; 126.8; 42.8.

## Benzamide


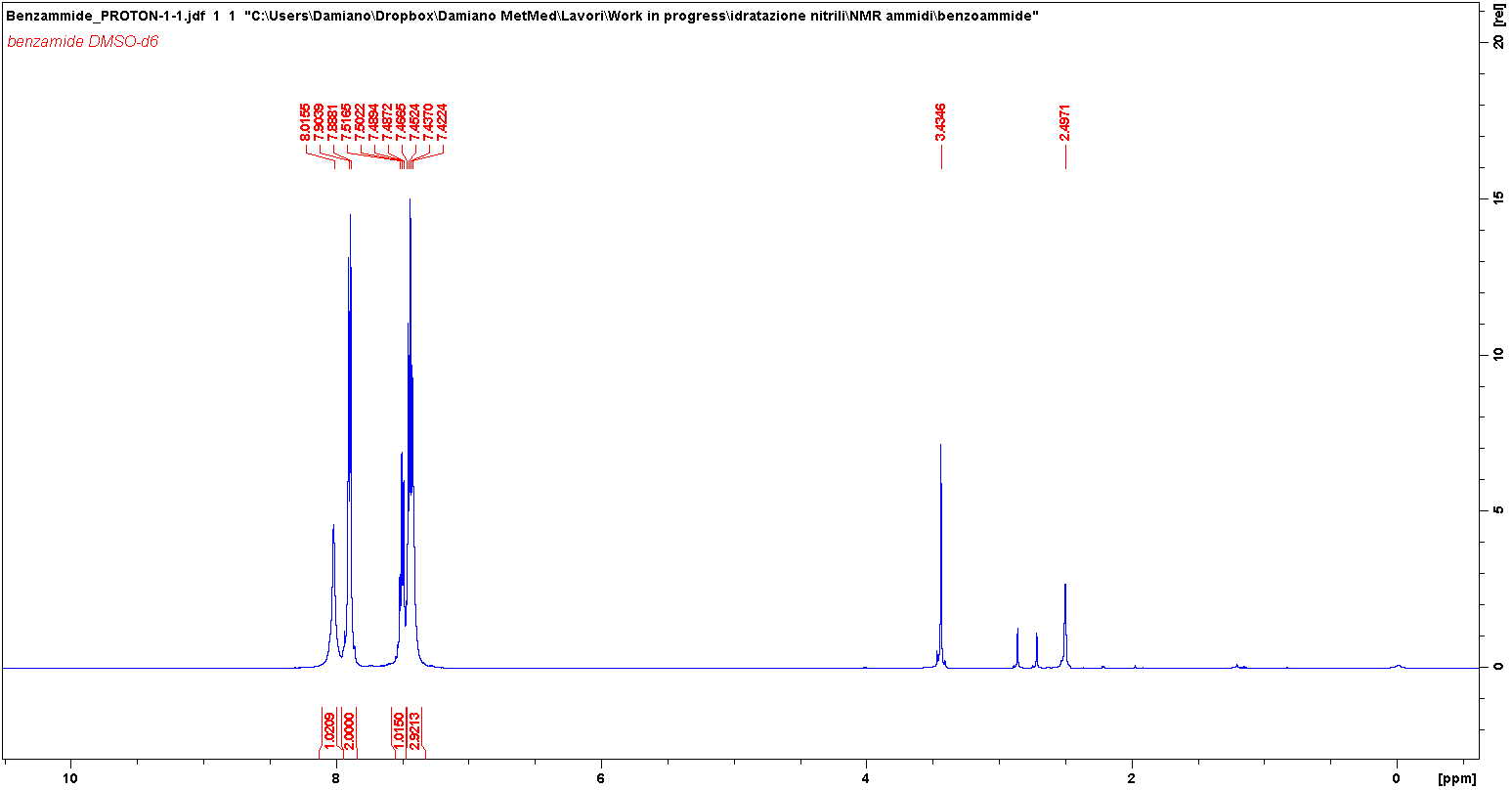


**Fig. S9.** Benzamide ^1^H NMR spectrum (500 MHz; DMSO-d_6_) δ: 8.01 (1H, b); 7.89 (2H, d, *J*= 7.90); 7.50 (1H, t, *J*= 7.32 Hz); 7.44 (3H, m).


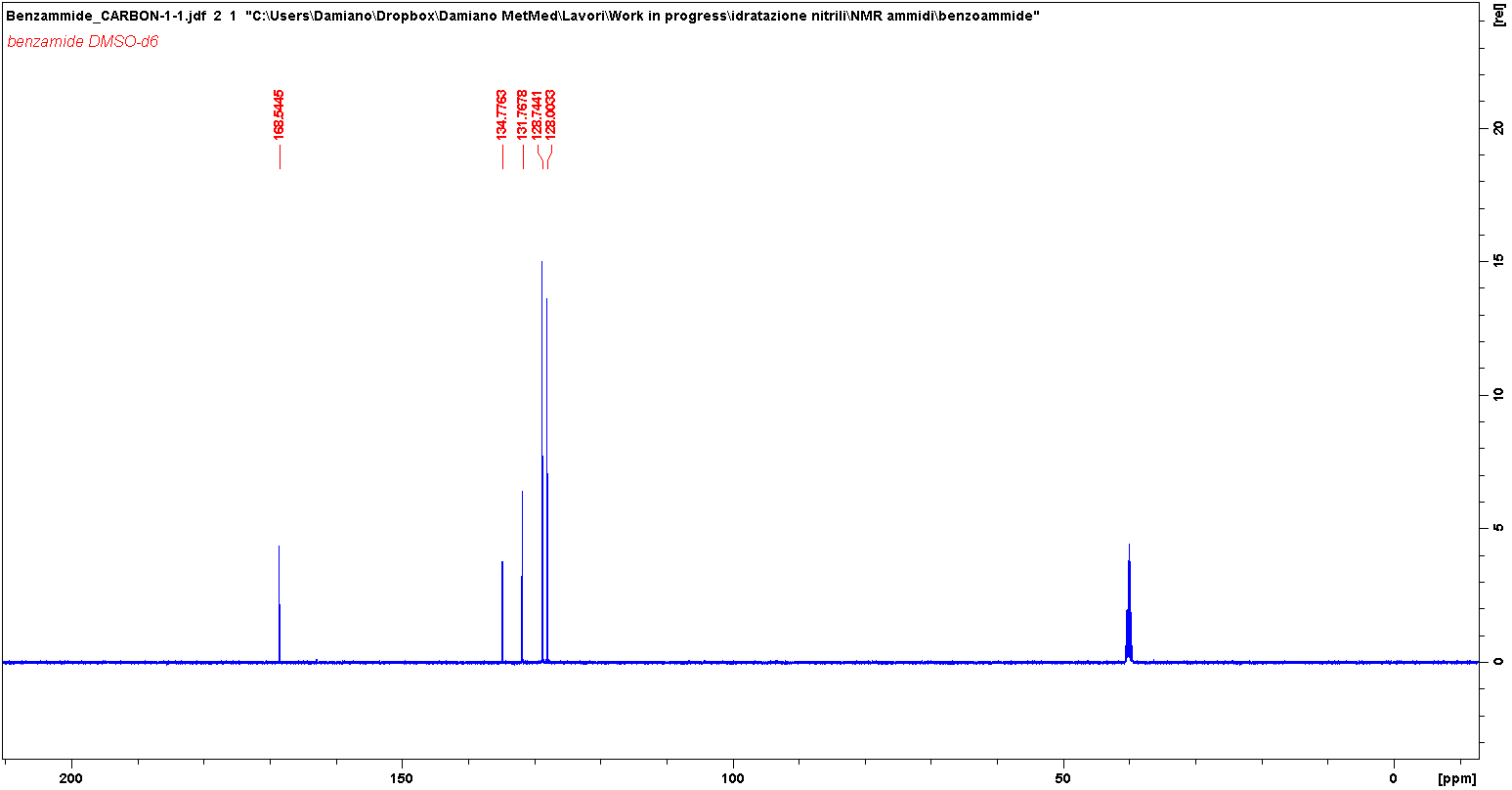


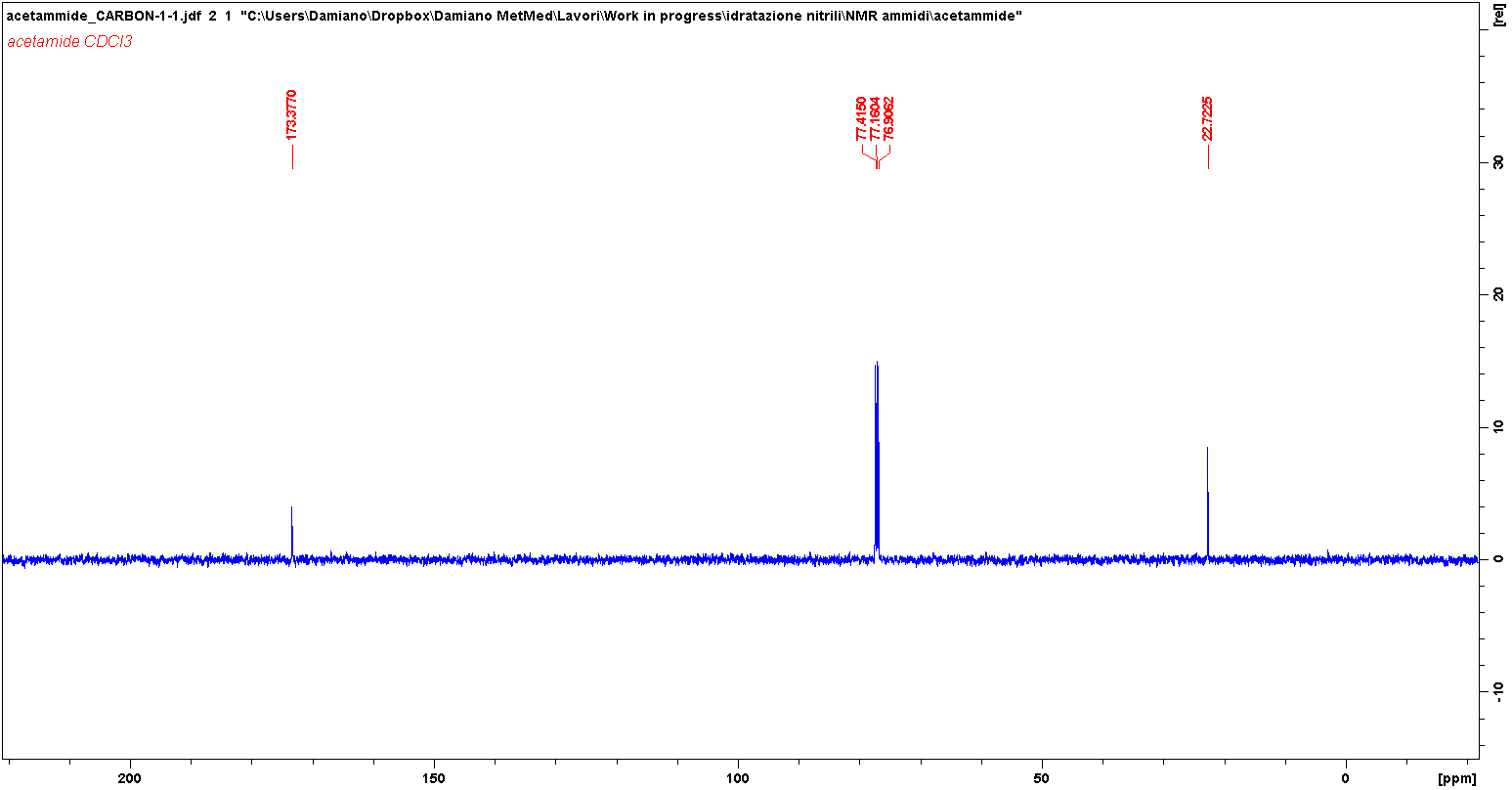
**Fig. S10.** Benzamide ^13^C NMR spectrum (125 MHz; DMSO-d_6_) δ: 168.5; 134.8; 131.8; 128.7; 128.0.

## 2-Hydroxybenzamide


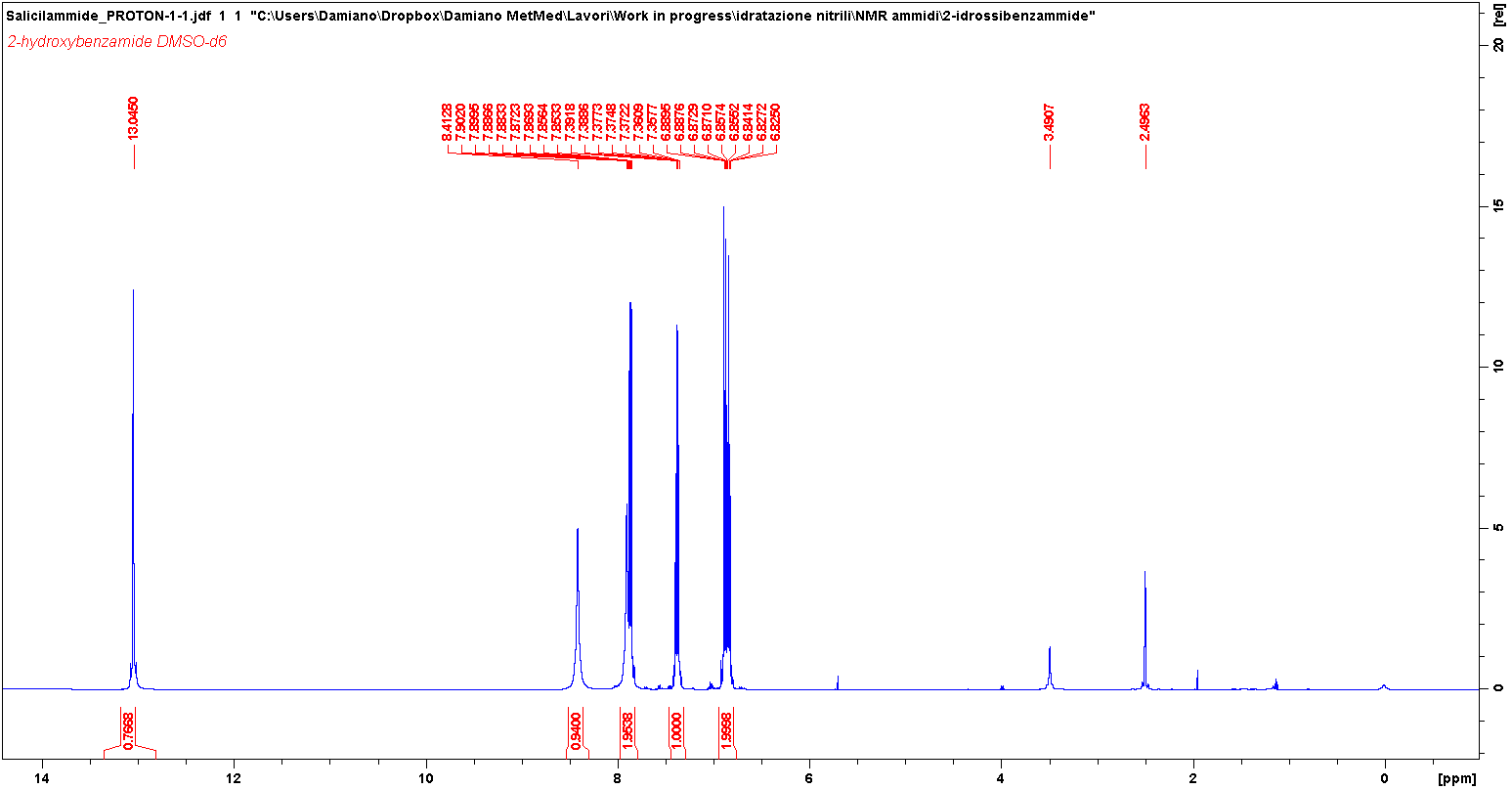


**Fig. S11.** 2-Hydroxybenzamide ^1^H NMR spectrum (500 MHz; DMSO-d_6_) δ: 13.04 (1H, s); 8.41 (1H, b); 7.90 (1H, b); 7.86 (1H, dd, *J_3_=* 7.96 Hz, *J_4_=* 1.53 Hz); 7.37 (1H, m); 6.88 (1H, dd, *J_3_=* 8.29 Hz, *J_4_=* 0.94 Hz); 6.84 (1H, m).


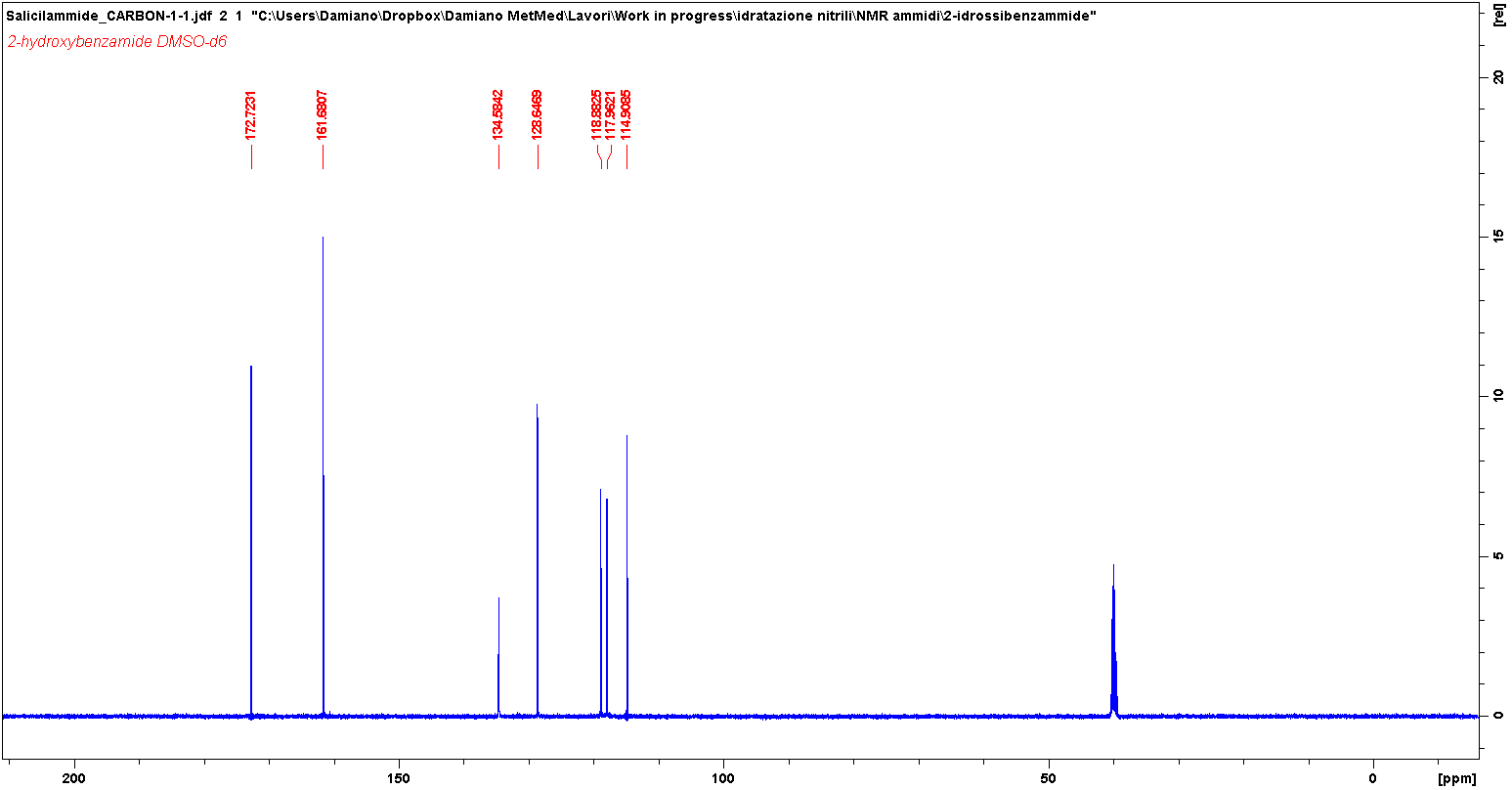


**Fig. S12.** 2-Hydroxybenzamide ^13^C NMR spectrum (125 MHz; DMSO-d_6_) δ: 172.7; 161.7; 134.6; 128.6; 118.9; 117.9; 114.9.

## 4-Dimethylamidobenzamide


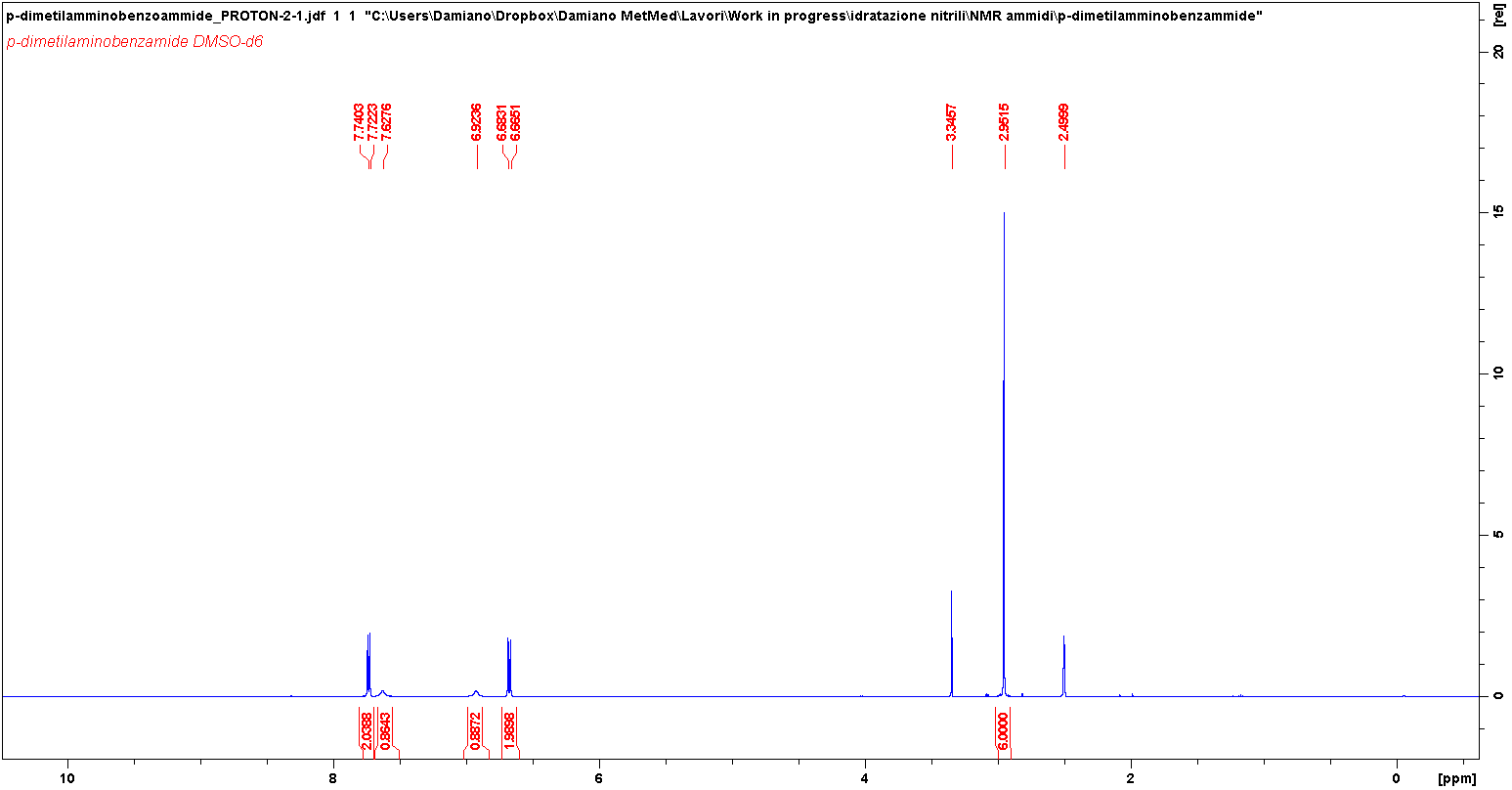


**Fig.S13.** 4-Dimethylaminobenzamide ^1^H NMR spectrum (500 MHz; DMSO-d_6_) δ: 7.73 (2H, d, *J*= 8.99 Hz); 7.63 (1H, b); 6.92 (1H, b); 6.67 (2H, d, *J*= 8.99 Hz); 2.95 (6H, s).


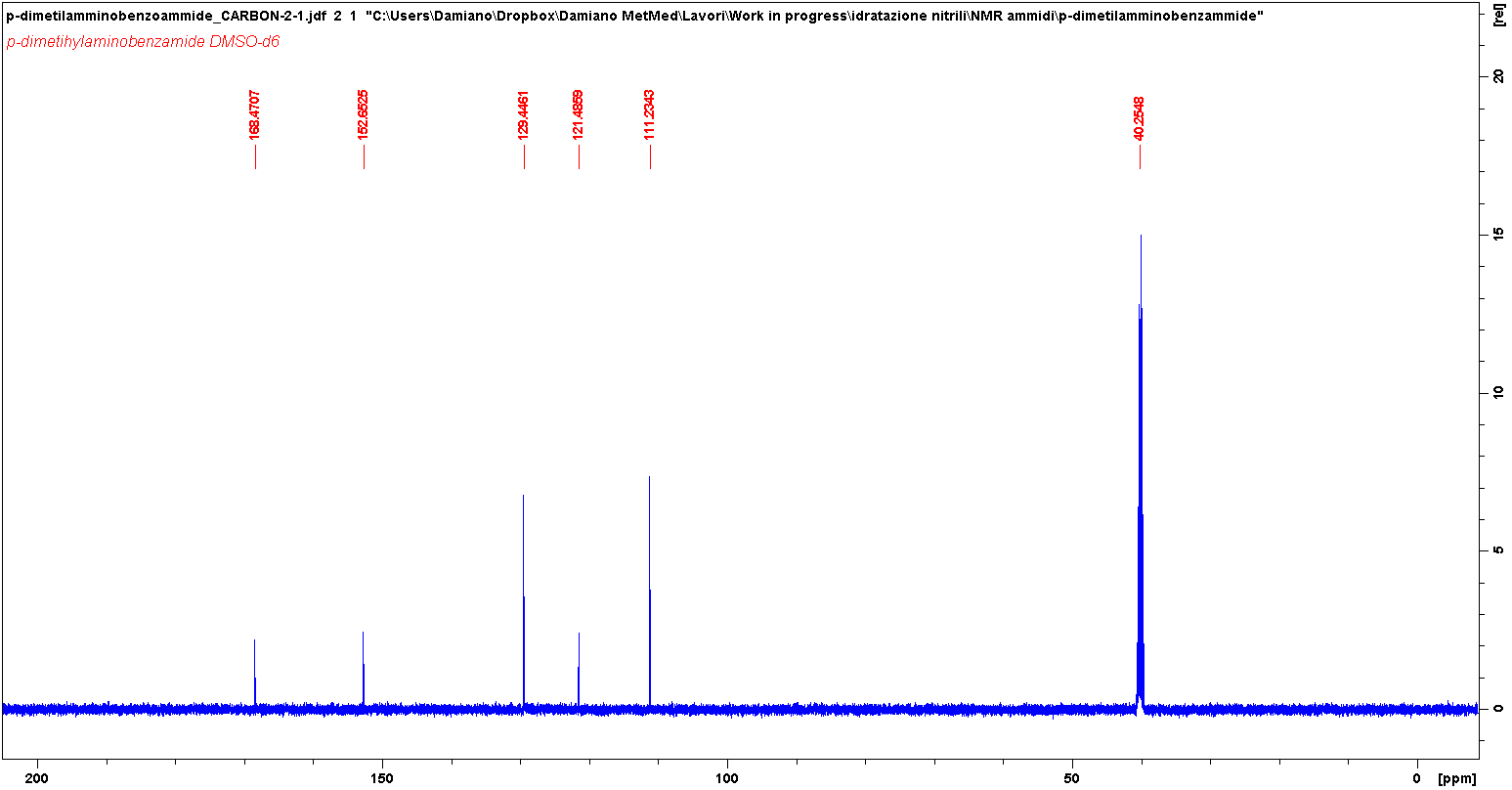


**Fig. S14.** 4-Dimethylaminobenzamide ^13^C NMR spectrum (125 MHz; DMSO-d_6_) δ: 168.5; 152.6; 129.4; 121.5; 111.2.

## 4-Bromobenzamide


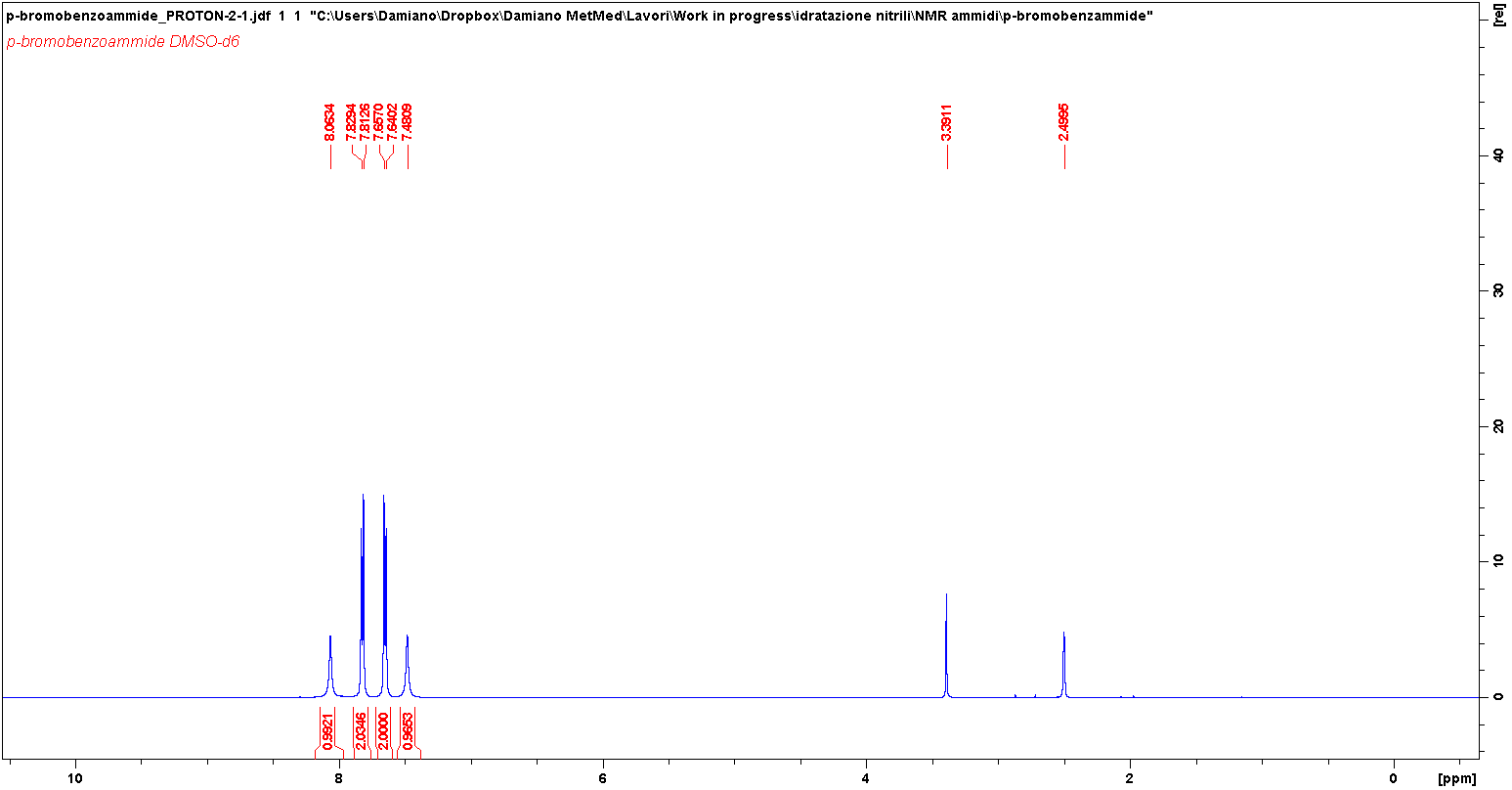


**Fig. S15.** 4-Bromobenzamide ^1^H NMR spectrum (500 MHz; DMSO-d_6_) δ: 8.06 (1H, b); 7.82 (2H, d, *J*= 8.40 Hz); 6.65 (2H, d, *J*= 8.40 Hz); 7.48 (1H, b).


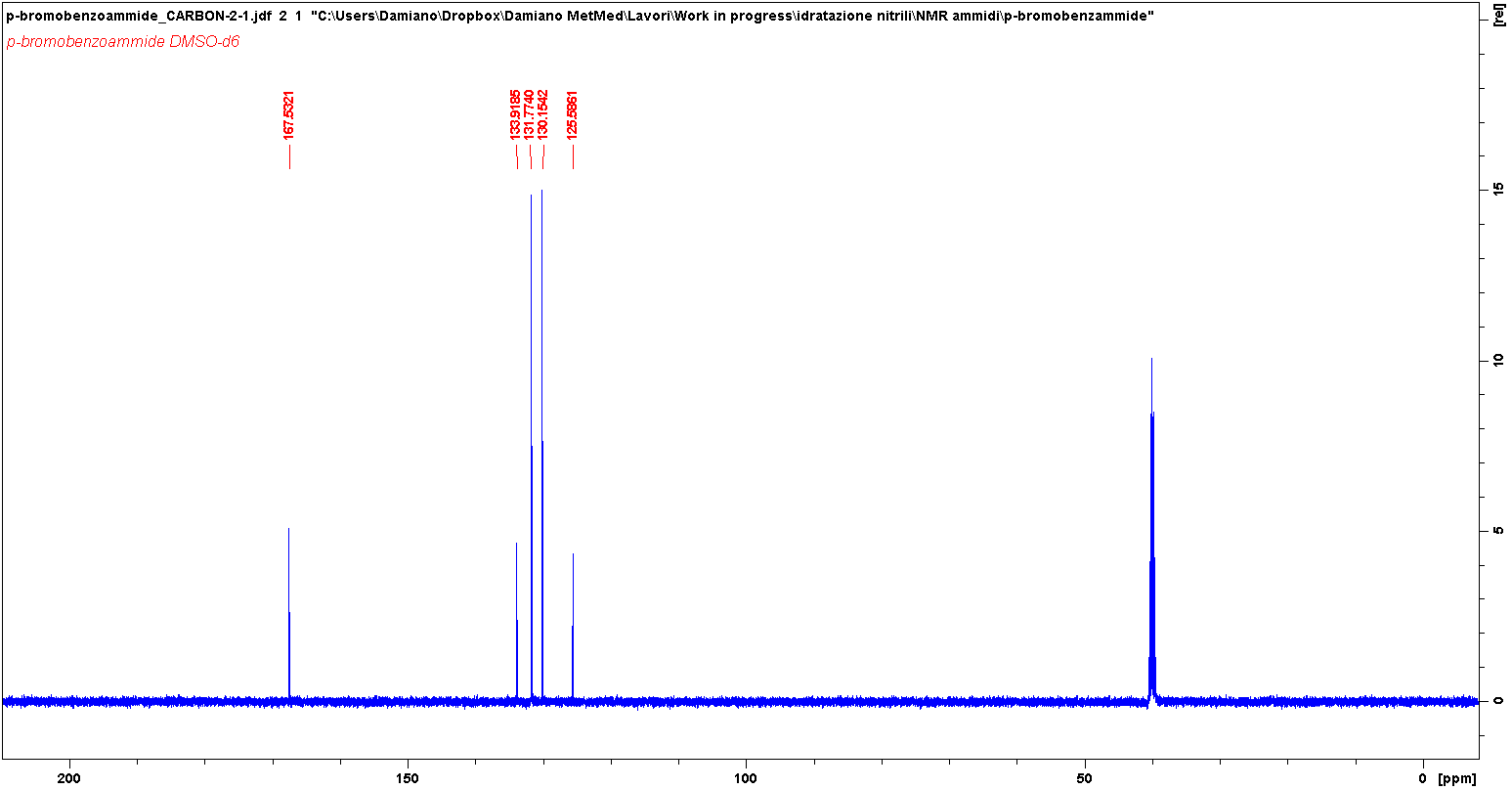


**Fig. S16.** 4-Bromobenzamide ^13^C NMR spectrum (125 MHz; DMSO-d_6_) δ: 167.5; 133.9; 131.8; 130.2; 125.6.

## 4-Nitrobenzamide


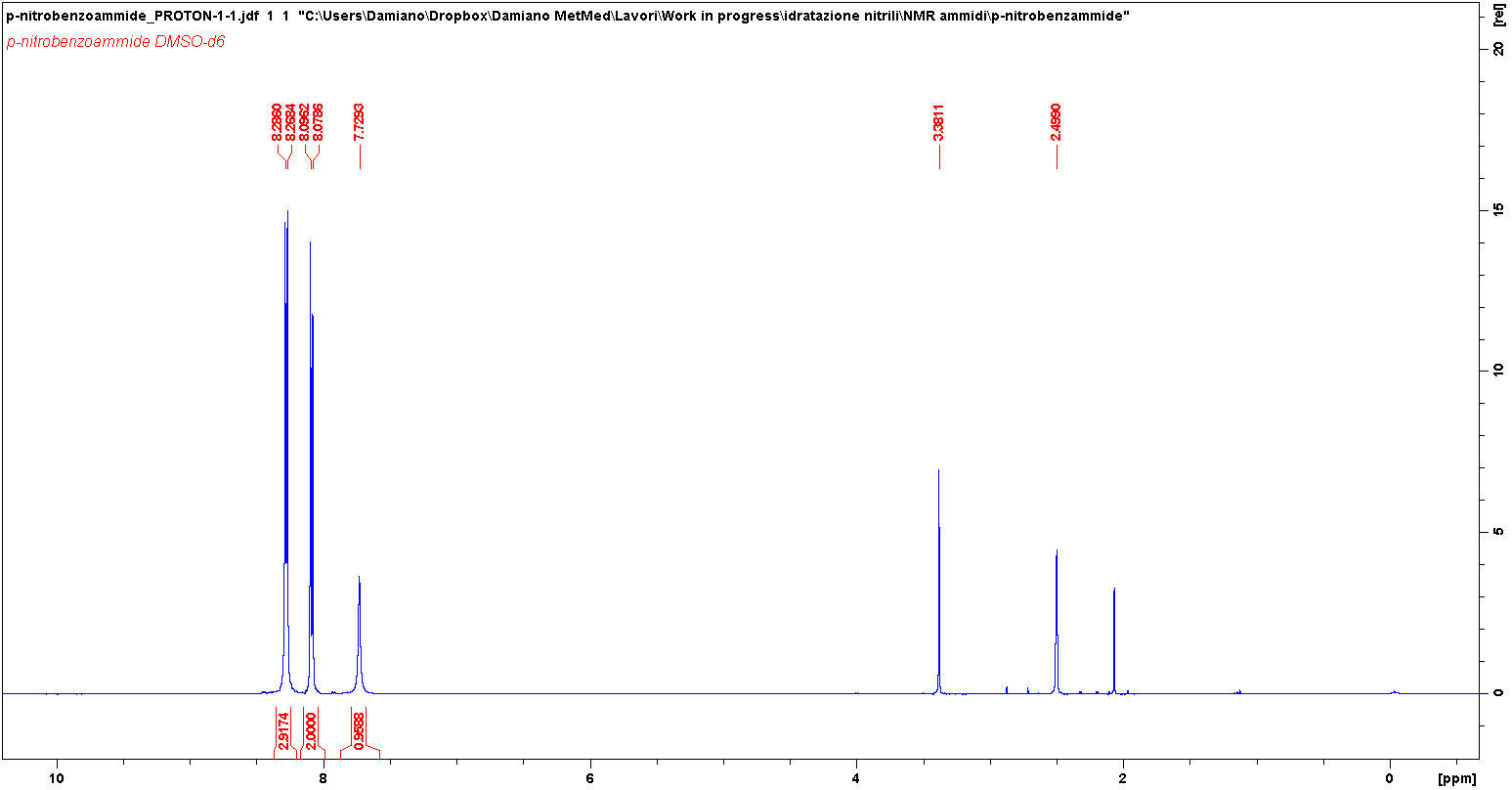


**Fig. S17.** 4-Nitrobenzamide ^1^H NMR spectrum (500 MHz; DMSO-d_6_) δ: 8.28 (3H, m); 8.09 (2H, d, *J*= 8.80 Hz).


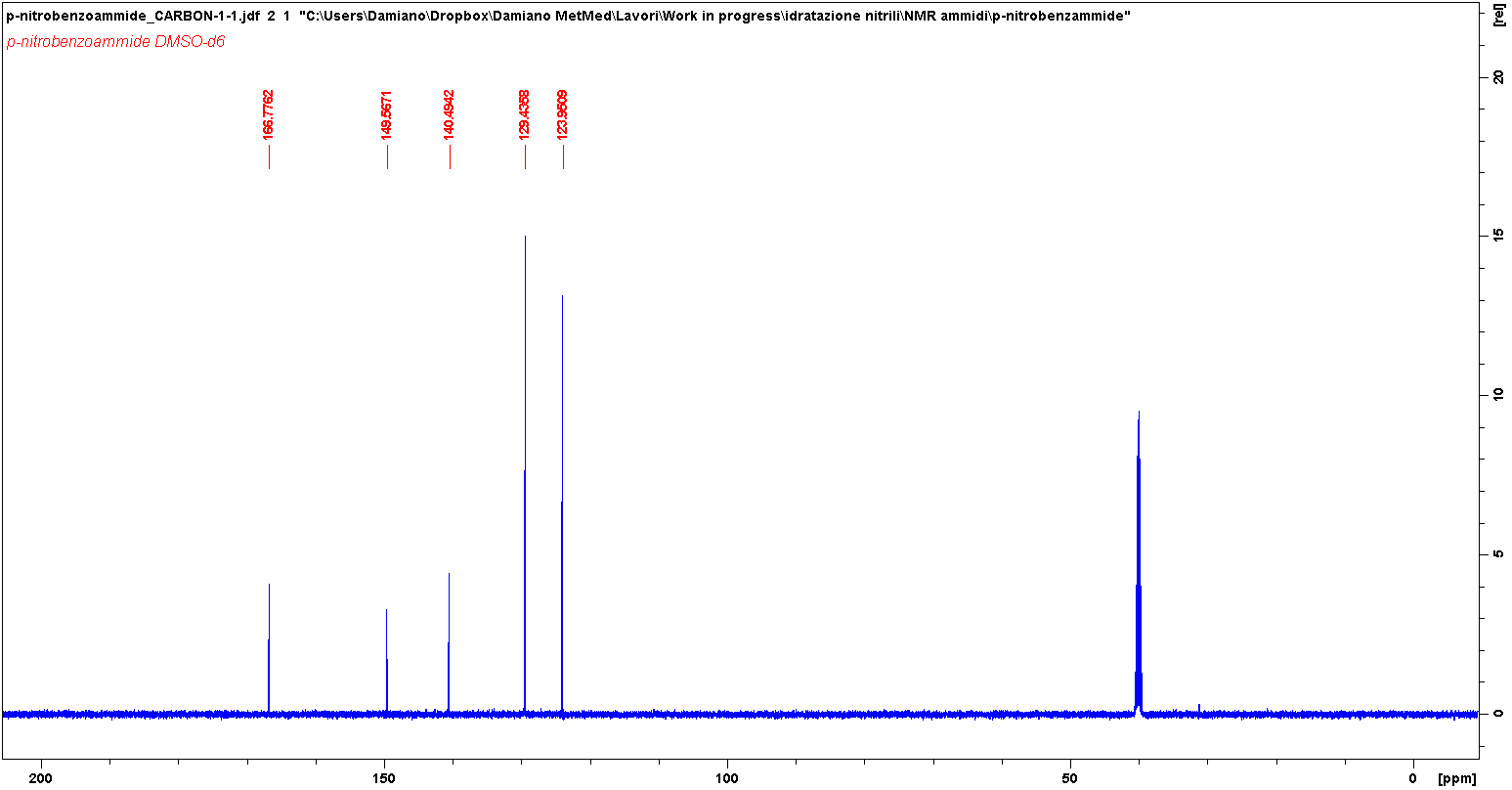


**Fig. S18.** 4-Nitrobenzamide ^13^C NMR spectrum (125 MHz; DMSO-d_6_) δ: 166.8; 149.6; 140.5; 129.4; 123.9.

## 2-Cyanobenzamide


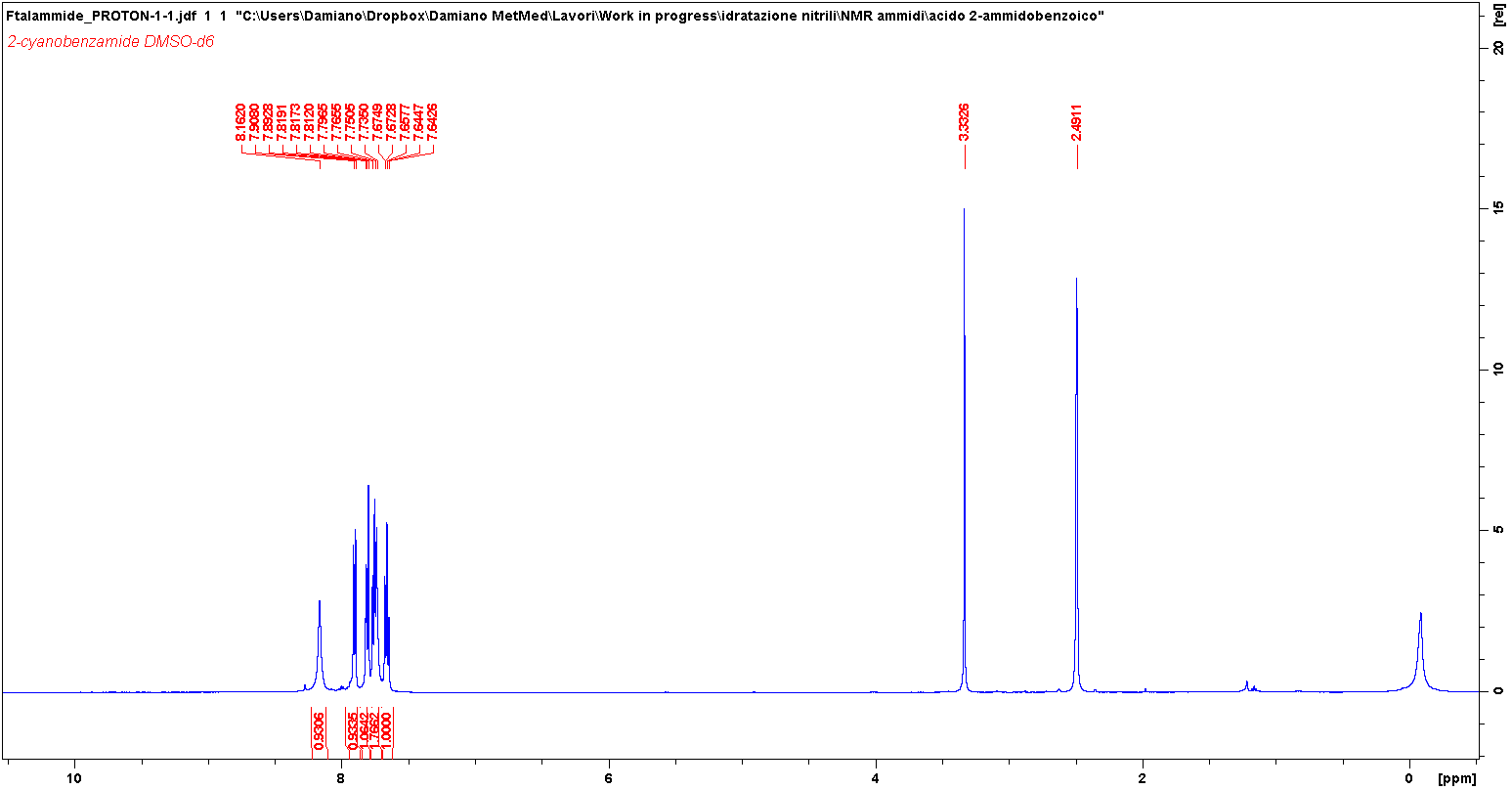


**Fig. S19.** 2-Cyanobenzamide ^1^H NMR spectrum (500 MHz; DMSO-d_6_) δ: 8.16 (1H, b); 7.90 (1H, d, *J*= 7.63 Hz); 7.80 (1H, m); 7.75 (2H, m); 7.66 (1H, m).


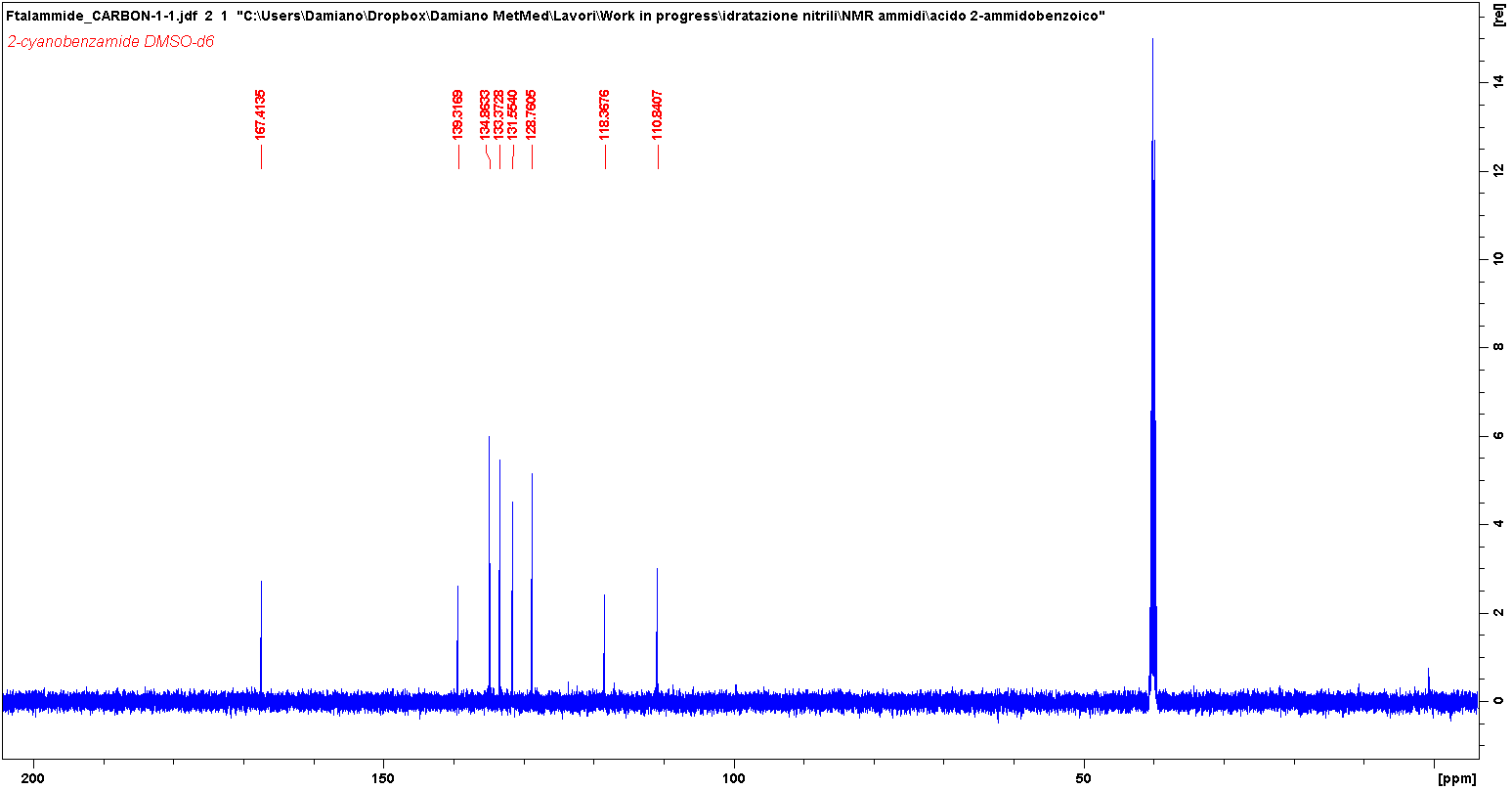


**Fig. S20.** 2-Cyanobenzamide ^13^C NMR spectrum (125 MHz; DMSO-d_6_) δ: 167.4; 139.3; 134.9; 133.4; 131.6; 128.8; 118.4; 110.8.

## 4-Cyanobenzamide


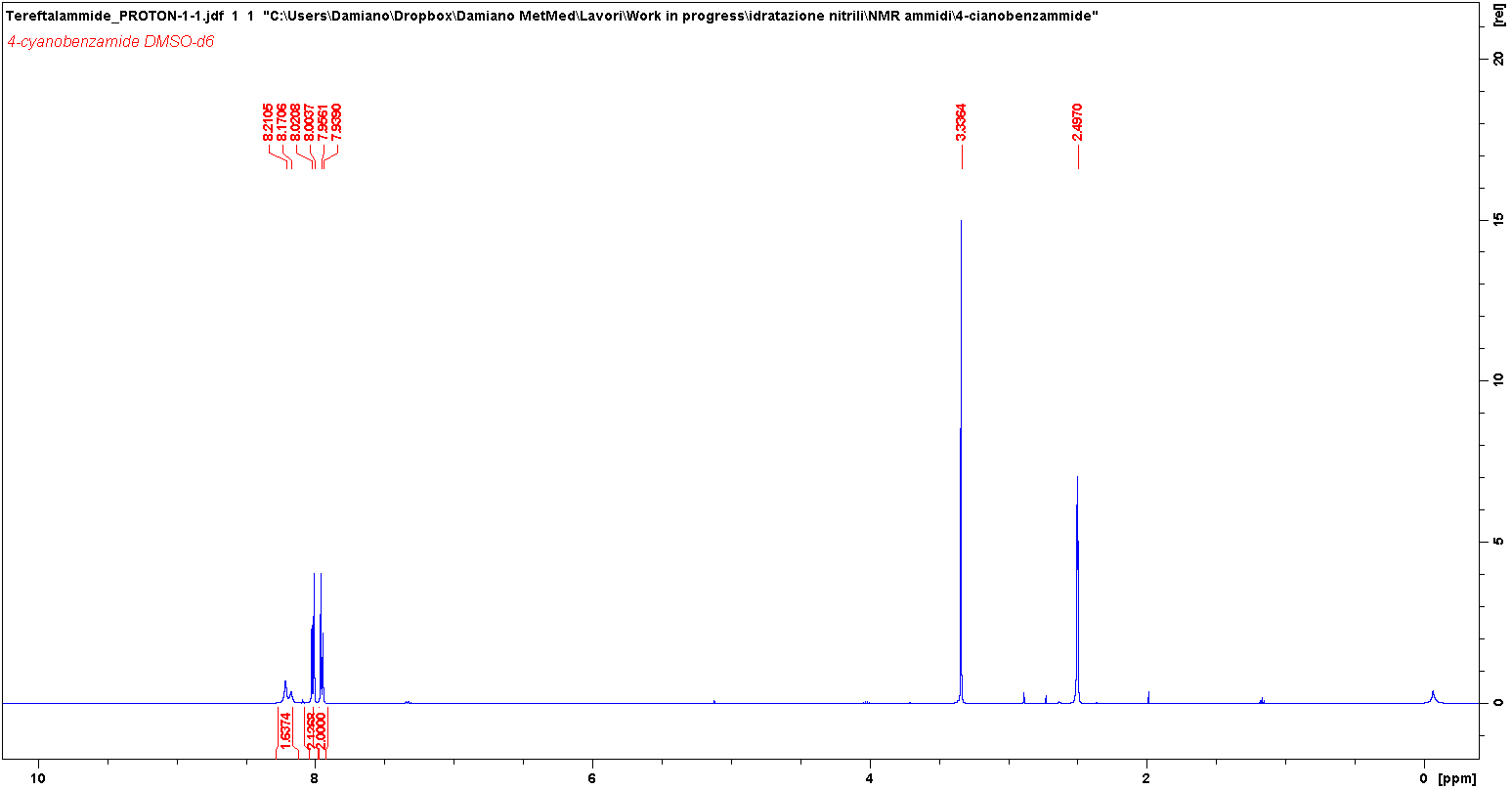


**Fig. S21.** 4-Cyanobenzamide ^1^H NMR spectrum (500 MHz; DMSO-d_6_) δ: 8.21 (1H, b); 8.17 (1H, b); 8.01 (2H, d, *J*= 8.56 Hz); 7.95 (2H, d, *J*= 8.56 Hz).


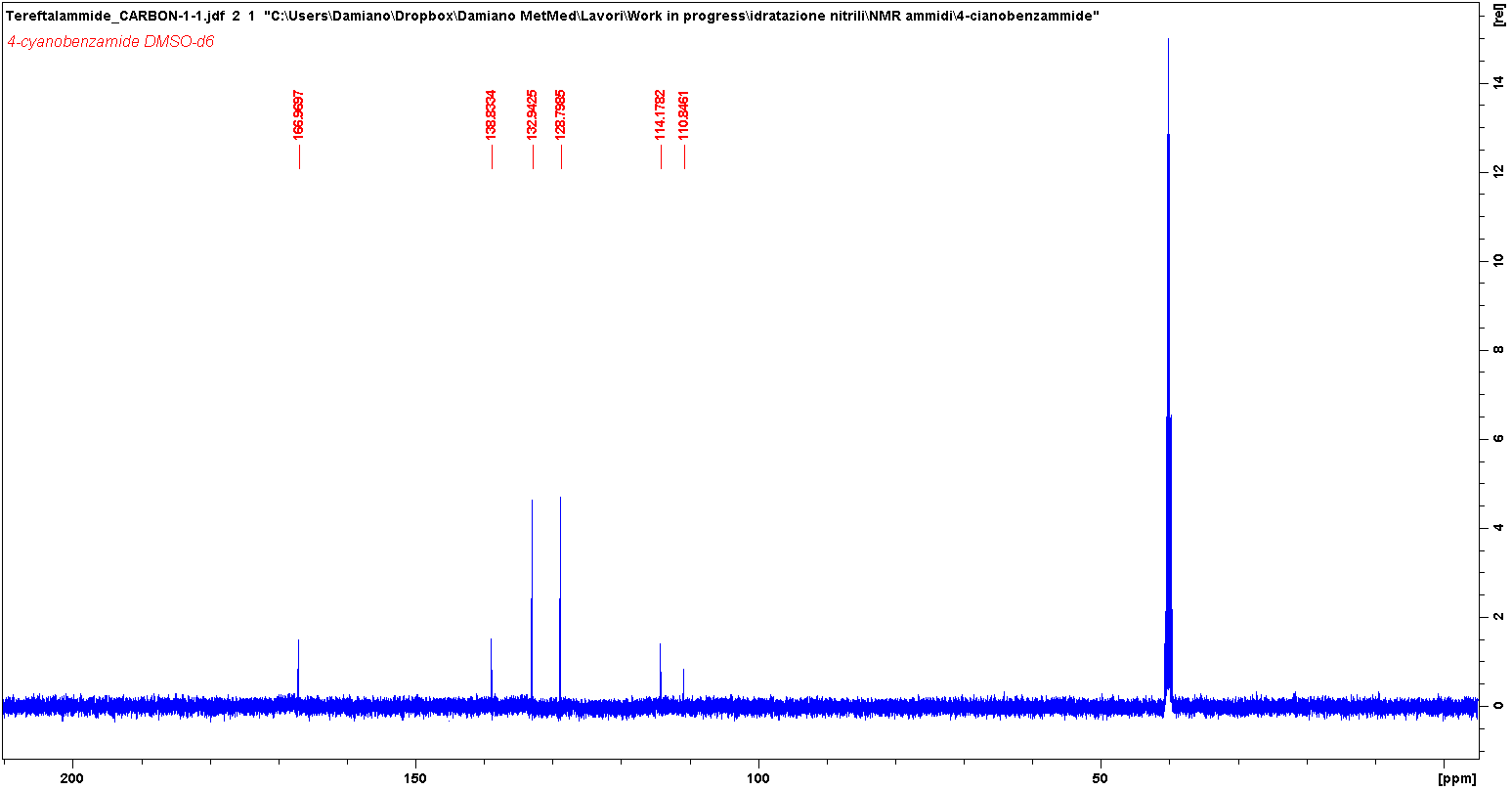


**Fig. S22.** 4-Cyanobenzamide ^13^C NMR spectrum (125 MHz; DMSO-d_6_) δ: 166.9; 138.8; 132.9; 128.8; 114.2; 110.8; 118.4; 110.8.

## 4-Carbamoyl-1-methylpyridin-1-ium nitrate


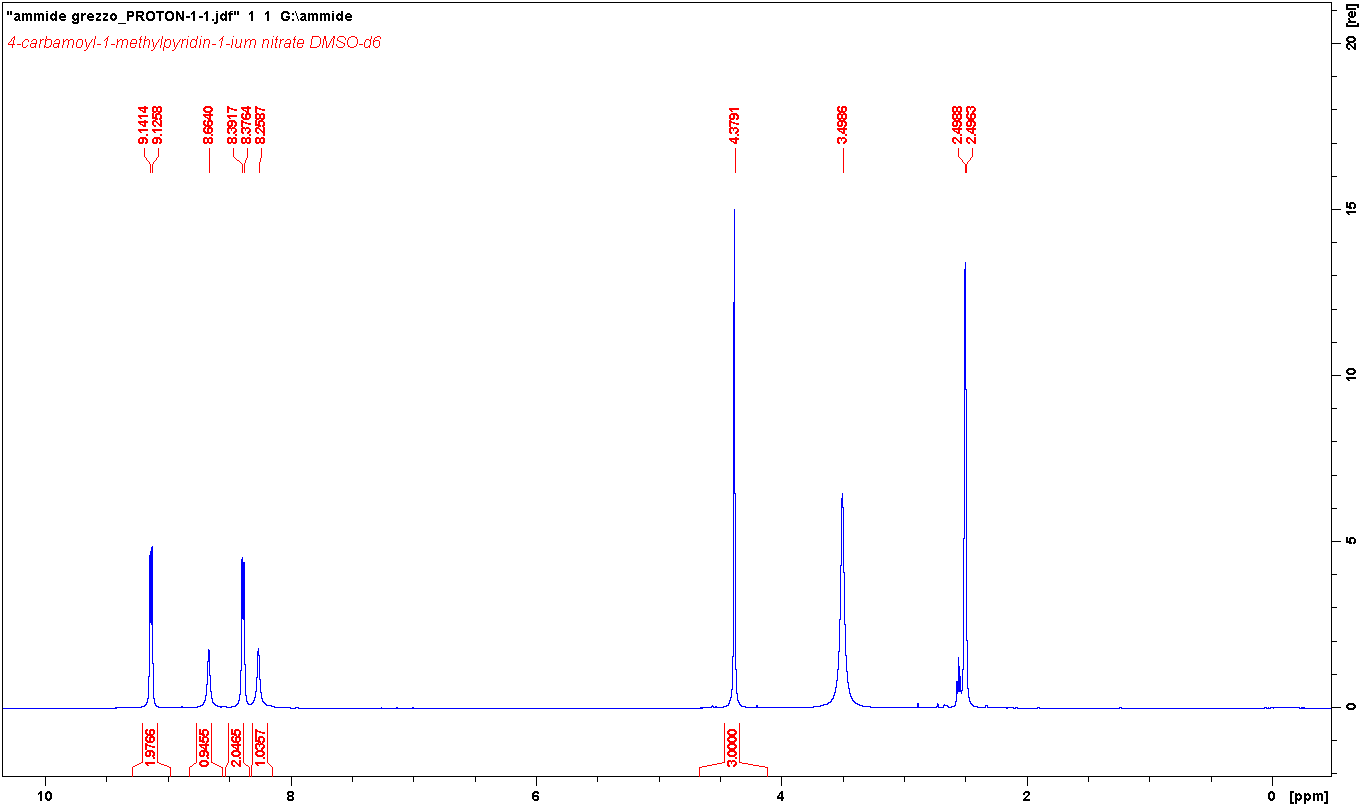


**Fig. S23.** 4-carbamoyl-1-methylpyridin-1-ium nitrate ^1^H NMR spectrum (500 MHz; DMSO-d_6_) δ: 9.13 (2H, d, *J*= 6.26 Hz); 8.66 (1H, b); 8.38 (2H, d, *J*= 6.12 Hz); 8.26 (1H, b).


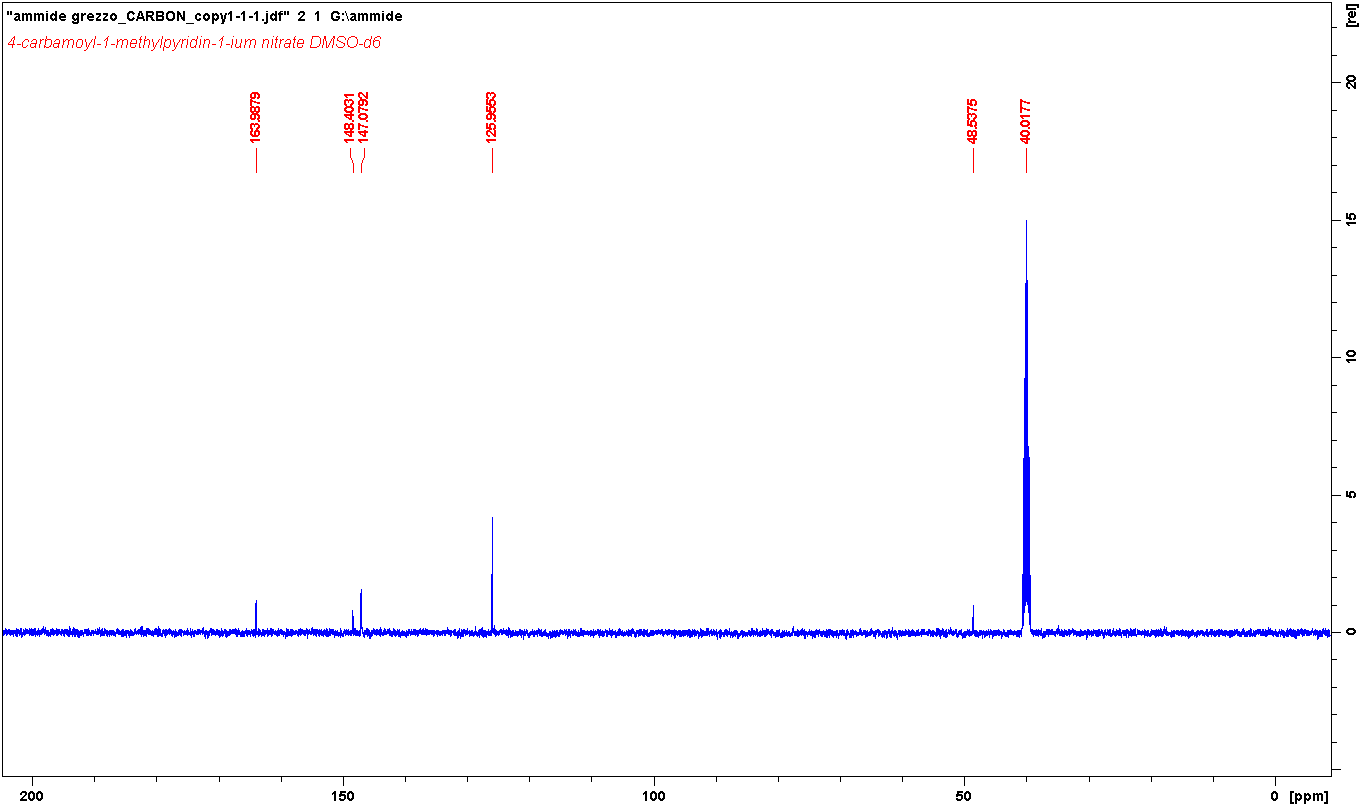


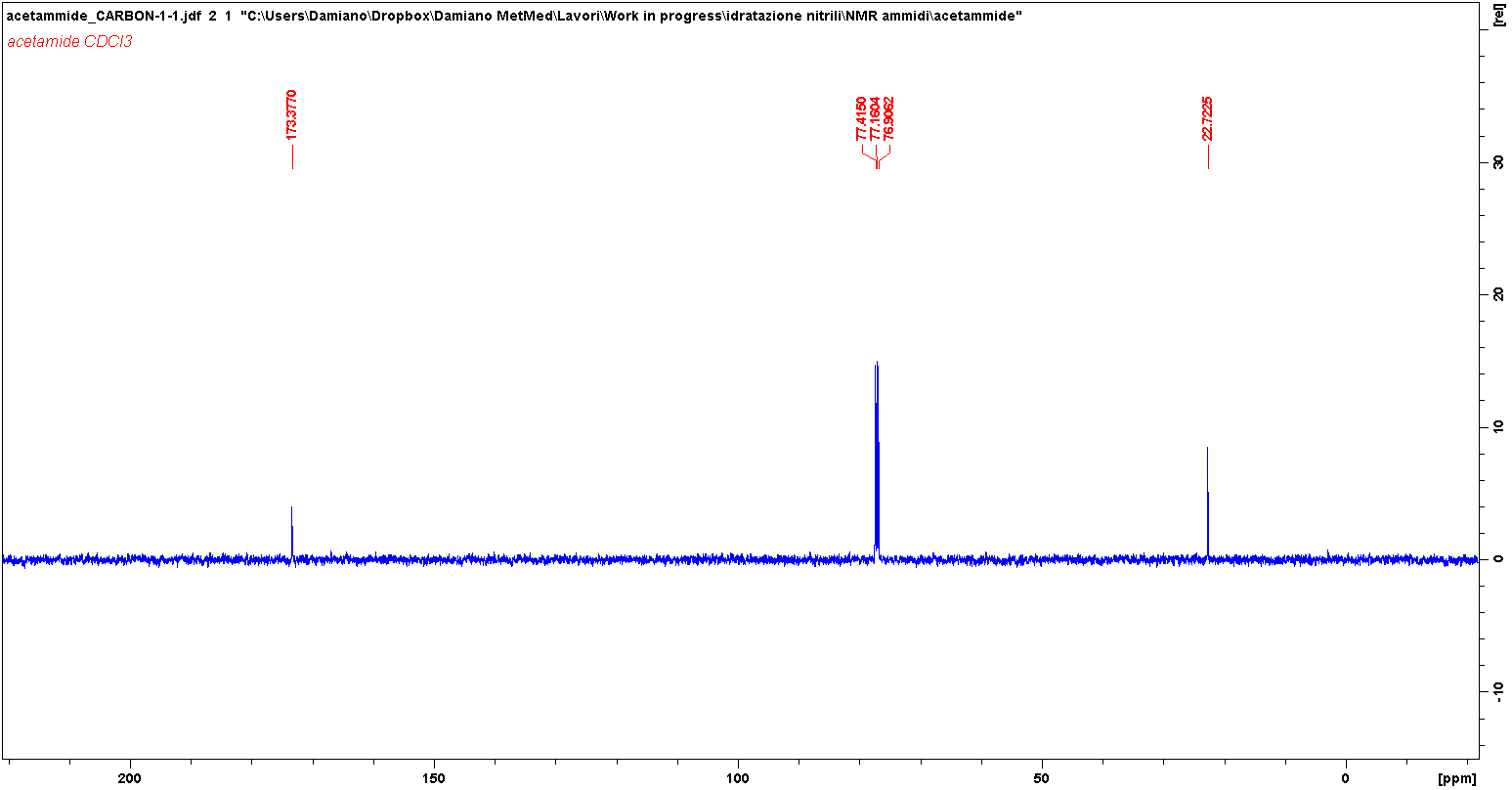
**Fig. S24.** 4-carbamoyl-1-methylpyridin-1-ium nitrate ^13^C NMR spectrum (125 MHz; DMSO-d_6_) δ: 163.9; 148.4; 147.1; 125.9; 48.5.
